# Exploring decarbonization pathways for USA passenger and freight mobility

**DOI:** 10.1038/s41467-023-42483-0

**Published:** 2023-10-30

**Authors:** Christopher Hoehne, Matteo Muratori, Paige Jadun, Brian Bush, Arthur Yip, Catherine Ledna, Laura Vimmerstedt, Kara Podkaminer, Ookie Ma

**Affiliations:** 1 National Renewable Energy Laboratory, Golden, CO USA; 2 U.S. Department of Energy, Washington, D.C. USA

**Keywords:** Energy modelling, Energy infrastructure, Computational science, Climate-change mitigation, Civil engineering

## Abstract

Passenger and freight travel account for 28% of U.S. greenhouse gas (GHG) emissions today. We explore pathways to reduce transportation emissions using NREL’s TEMPO model under bounding assumptions on future travel behavior, technology advancement, and policies. Results show diverse routes to 80% or more well-to-wheel GHG reductions by 2050. Rapid adoption of zero-emission vehicles coupled with a clean electric grid is essential for deep decarbonization; in the median scenario, zero-emission vehicle sales reach 89% for passenger light-duty and 69% for freight trucks by 2030 and 100% sales for both by 2040. Up to 3,000 terawatt-hours of electricity could be needed in 2050 to power plug-in electric vehicles. Increased sustainable biofuel usage is also essential for decarbonizing aviation (10–42 billion gallons needed in 2050) and to support legacy vehicles during the transition. Managing travel demand growth can ease this transition by reducing the need for clean electricity and sustainable fuels.

## Abstract (web summary)

Rapid adoption of zero-emission vehicles with a concurrent transition to clean electricity is essential to achieve U.S. transportation decarbonization goals. Managing travel demand can ease this transition by reducing the need for clean electricity supply. @cghoehne, @nrel, #NRELMobility

## Introduction

After more than a century of petroleum dominance, the transportation sector is on the verge of radical transformations driven by rapid technology advancement of alternative fuels and powertrains, emerging mobility options and business models, and increased ambitions at all levels of governance to tackle climate change and improve air quality. However, the future of mobility and its role in climate change mitigation remains uncertain and controversial. Much work is still needed to identify the extent and speed in which the transportation sector can decarbonize and to determine what mix of technologies and policies can best support a sustainable transition.

Historically, transportation was expected to play a relatively limited role in reducing greenhouse gas (GHG) emissions despite consistently accounting for over a quarter of annual GHGs^1^. The Intergovernmental Panel on Climate Change’s Fifth Assessment Report^2^ stated, “Reducing transport emissions will be a daunting task given the inevitable increases in demand and the slow turnover and sunk costs of stock… and infrastructure.” The 2016 U.S. Mid-Century Strategy envisioned a pathway to achieve 80% economy-wide GHG emissions reductions by 2050 from 2005 despite limited emissions reductions from transportation, with petroleum providing over half of transportation energy use and low-carbon electricity providing only a quarter^3^. In these scenarios, carbon dioxide removal is relied upon to compensate for limited end-use emissions reductions. Even more recently, results from over 15 models participating in the 37th Stanford Energy Modeling Forum’s study on net-zero emissions indicate that transportation and industry sectors have the largest variation in emissions pathways across models, and residual emissions from transportation in net-zero scenarios are typically the largest of any sector^4^.

Some recent trends, however, point to much greater opportunities for affordable and sustainable options to enable and accelerate transportation decarbonization. This is driven by recent progress in electric vehicle (EV) and other clean fuel technologies and revised thinking that structural mobility changes will be achievable in the future^5,6^. Key strategies to achieve transportation decarbonization include vehicle electrification (supported by a decarbonized electricity grid), increased use of public transit and active travel modes, improved urban planning, efficient and intelligent operations, low- or zero-carbon fuels (e.g., sustainable biofuels, hydrogen), and supportive coordinated policies at all levels of government^7^. Many recent decarbonization scenarios are projecting aggressive transportation energy and emissions reductions by 2050 by combining such strategies^8–11^. For example, in the International Energy Agency (IEA) “Net-Zero by 2050” scenario^8^, transportation emissions are reduced by 90% between 2020 and 2050 despite rapidly growing passenger and freight activity. These reductions are enabled by a mix of solutions including policies to promote mode shifts, improvements in vehicle efficiency and systems operations, use of low-carbon fuels, and widespread electrification. Widespread electrification provides large carbon emissions reductions with >60% of global light-duty vehicle (LDV) sales (75% in advanced economies) electrified by 2030, with EVs reaching 90% of sales in 2035 and the remaining 10% being hydrogen vehicles. Similar levels of emissions reductions are shown in the 2021 Long-Term Strategy of the United States for a net-zero-emission economy by 2050, with scenarios including 80%–100% direct transportation emissions reductions^12^. The Princeton Net-Zero America project^10^ also identifies pathways to reduce U.S. transportation energy demand by 30%–50% by 2050, with reductions in energy use for every travel mode except aviation. The study points to light-duty EV sales of 60%–100% in the 2040s and 50%–100% medium and heavy truck sales being a combination of EVs and fuel cell electric vehicle (FCEVs).

Despite growing interest in deep emissions reductions in transportation, and some optimistic projection from economy-wide integrated models, the pathways to achieve ambitious goals remain uncertain, and there is skepticism in the efficacy of some decarbonization strategies^13–15^. For example, improved fuel efficiency can have negative feedbacks to the effectiveness of mode shifting and alternative fuel adoption^16,17^, and many studies ultimately find that shifting behavior and reducing travel demand will be necessary to achieve significant sector decarbonization^8,18–22^. Milovanoff et al.^20^ evaluate current U.S. polices and find that LDV electrification alone is not sufficient to hit carbon mitigation targets consistent with preventing 2 °C warming and point to a need for a wide range of policies, including measures to reduce vehicle ownership and usage. However, a transition away from private vehicle dependence in the United States would be very difficult, as Moody et al.^23^ find that over half of the value in owning a private car is for control over travel schedule, reliability, and flexibility of travel. There are also conflicting outcomes for shared mobility services: Several studies have found that shared mobility services are likely to worsen travel congestion due to deadheading and induced trips^24–27^, but other research has demonstrated emissions reductions for certain applications^28–30^.

Currently, few high-resolution models exist that capture the transportation system collectively with the ability to represent new technology and mobility solutions to inform decision makers and investments^31^. To address these gaps and surrounding uncertainty, we use the Transportation Energy & Mobility Pathway Options^TM^ (TEMPO)^32^ system model to simulate the possible evolution of emissions from passenger and freight mobility in the United States to 2050. The TEMPO model is novel in its focus and ability to represent new clean energy technologies and model important mobility nuances and consumer heterogeneities while offering a national systems-level perspective^31,32^. We explore the evolution of future transportation energy use and emissions by simulating 2173 long-term scenarios focusing on the impacts of different technologies, fuels, policies, and changes in travel behavior and choices guided by expert elicitation. This allows us to study the underlying uncertainties associated with complex system-level mobility evolution by isolating various levers of change (e.g., the impact of battery cost reductions) and by combining dozens of drivers of potential futures (e.g., what combinations of assumptions lead to the best outcomes for decarbonization). This approach enables identifying the overall drivers of sector-wide emissions changes and the effects of broad efforts with greater detail than previously modeled to inform how deep decarbonization could be achieved.

## Results

### Sensitivity of input variables

We first report results on the univariate scenario design that quantifies and ranks the sensitivity of outcomes (well-to-wheel GHG emissions and zero-emission vehicle [ZEV] adoption) to input variables in isolation. We compare model results for the national baseline (aligned with the Annual Energy Outlook [AEO] reference case) to 172 scenarios in which one input was varied at a time and all others held constant to the baseline. In addition to reporting isolated impacts to 2050 U.S. passenger and freight mobility emissions, we also report isolated impacts to ZEV stocks, as ZEVs are poised to be key technologies to decarbonize on-road travel, which currently accounts for over 80% of U.S. mobility GHGs^33^.

**Fig. 1:**
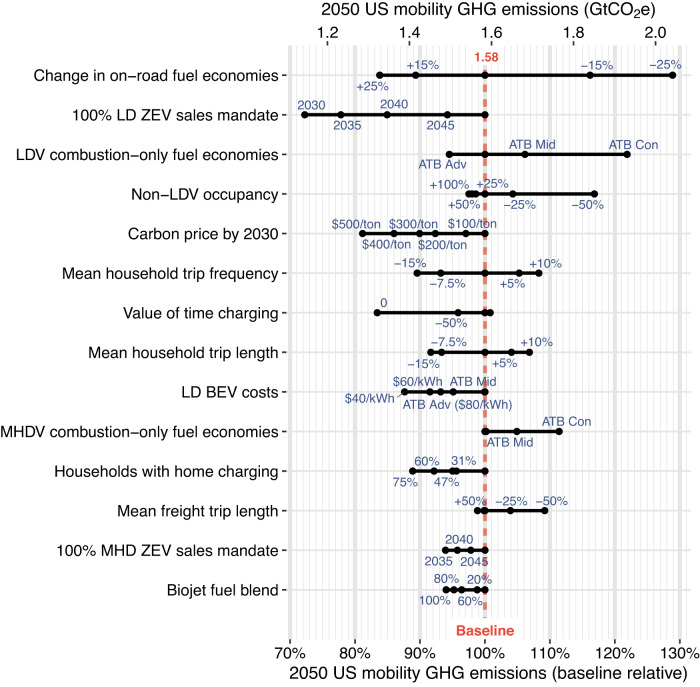
Isolated variable impacts on well-to-wheel U.S. mobility greenhouse gas (GHG) emissions in 2050. Input categories (y-axis) are ranked by greatest to least absolute impact. Input categories with maximum emissions impacts <5% in 2050 are excluded (*N* = 37). Carbon prices are per metric ton CO_2_e. Battery electric vehicle (BEV) costs and fuel economies are based on the 2020 Annual Technology Baseline (ATB) study^89^, with ATB “Con,” “Mid,” and “Adv” referring to the constant, mid, and advanced scenarios, respectively, with two additional scenarios of battery cost reduction assumptions ($60 kWh^−1^ and $40 kWh^−1^ by 2050). LD light duty, LDV light-duty vehicle, MHD medium-/heavy-duty, MHDV medium-/heavy-duty vehicle, ZEV zero-emissions vehicle.

In the baseline scenario, emissions are reduced by 24% relative to 2019, and we show that further moderate reductions in overall mobility emissions can be achieved by several input variables in isolation (up to 28% relative to the 2050 baseline). For more details on the baseline scenario, see Supplementary Section 2. Figure 1 shows the isolated impacts on total passenger and freight GHG emissions relative to the 2050 baseline. Input variables are shown as black dots, and each input category is ranked by order of absolute impact. In isolation, the most impactful variable overall (top row) and the most impactful at increasing total mobility emissions is a decrease in on-road fuel economies: a 25% reduction in on-road fuel economies increases 2050 emissions by 0.46 Gt CO_2_e relative to the 2050 baseline (29% increase). The most impactful variable at reducing total mobility emissions is a policy mandating 100% light-duty ZEV sales by 2030, avoiding 0.44 Gt CO_2_e in 2050 relative to the 2050 baseline (28% reduction). Due to vehicle stock turnover, this policy would continue to reduce emissions past 2050. The magnitude of impacts from one mode or technology reflects the relative size of the various subsectors. For example, ZEV sales mandates for LDVs have greater total emissions reductions than medium-/heavy-duty vehicles (MHDVs) due to the larger share of emissions from LDVs, but when calculating the relative subsector impacts, they are comparable: For a 2035 mandate, a transition to light-duty ZEVs led to an isolated reduction of 0.35 Gt CO_2_e in 2050 (29% reduction from 2050 baseline passenger mobility emissions), while a transition to MHD ZEVs led to a reduction of 0.10 Gt CO_2_e in 2050 (a 26% reduction from baseline 2050 freight mobility emissions).

**Fig. 2:**
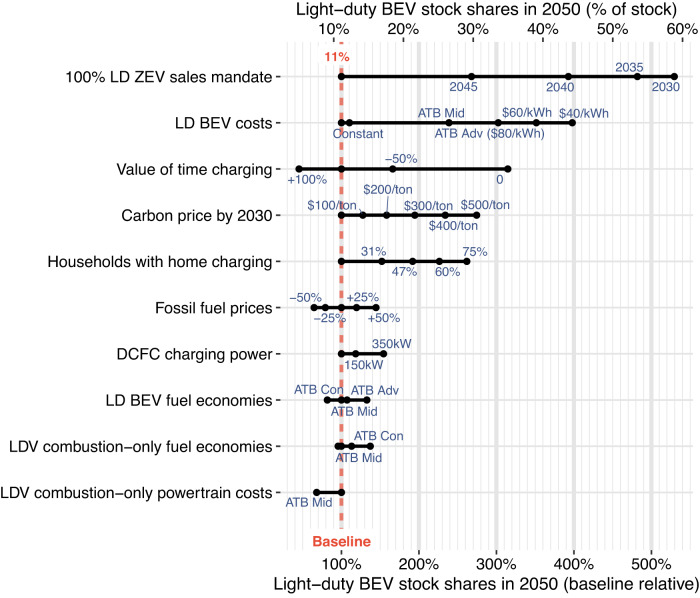
Isolated variable impacts on light-duty (LD) battery electric vehicle (BEV) stock shares in 2050. Input categories (y-axis) are ranked by greatest to least absolute impact. Scenarios with stock shares that changed <4.0% (absolute, not relative) from the baseline are excluded. For electricity price scenarios, “res” and “com” refer to residential and commercial prices, respectively. Carbon prices are per metric ton CO_2_e. BEV costs and fuel economies are based on the 2020 Annual Technology Baseline (ATB) study^89^, with ATB “Con,” “Mid,” and “Adv” referring to the constant, mid, and advanced scenarios, respectively, with two additional scenarios of battery cost reduction assumptions ($60 kWh^−1^ and $40 kWh^−1^ by 2050). LDV light-duty vehicle, ZEV zero-emissions vehicle, DCFC direct current fast charge.

**Fig. 3:**
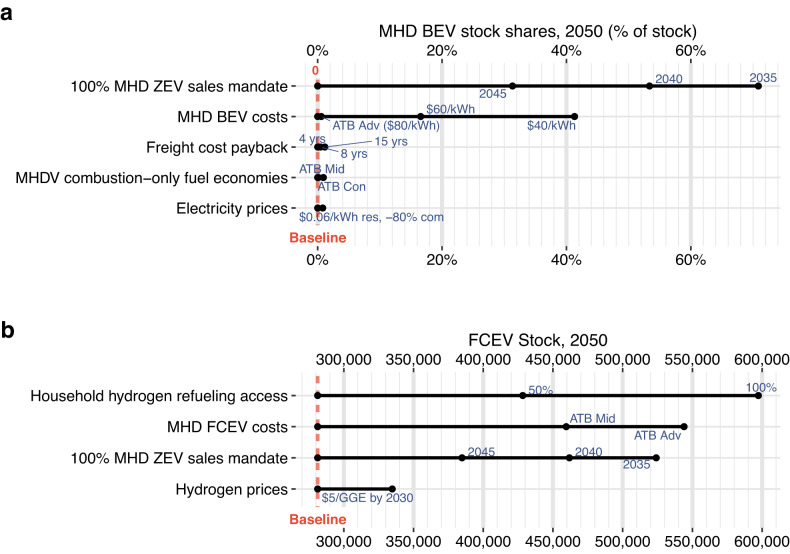
Isolated variable impacts on medium-/heavy-duty (MHD) battery electric vehicle (BEV) stock shares in 2050 and fuel cell electric vehicle (FCEV) stock (all duties) in 2050. Input categories (*y*-axis) are ranked by greatest to least absolute impact. Scenarios with MHD BEV stock shares that increased <50,000 in 2050 are excluded in (**a**) and all other omitted input categories in (**b**) had no impact to MHD stock in isolation. The baseline MHD stock shares in 2050 are 0.14% BEVs and 0% FCEVs. For electricity price scenarios, “res” and “com” refer to residential and commercial prices, respectively. BEV and FCEV costs and fuel economies are based on the 2020 Annual Technology Baseline (ATB) study^89^, with ATB “Con,” “Mid,” and “Adv” referring to the constant, mid, and advanced scenarios, respectively, with two additional scenarios of battery cost reduction assumptions ($60 and $40 kWh^−1^ by 2050). GGE gasoline gallon equivalent, ZEV zero-emissions vehicle.

We also explore the sensitivity of input variables on battery-electric vehicle (BEV) and FCEV stock shares in 2050 to understand the key drivers of ZEV adoption rates. Impacts to 2050 vehicle stock are shown for light-duty BEVs in Fig. 2, MHD BEVs in Fig. 3a, and MHD FCEVs in Fig. 3b. As expected, mandating 100% ZEV sales is the most impactful variable on BEV stock (top rows in Fig. 2 and Fig. 3a). For LDVs, a 2030 ZEV sales mandate results in 81% EV stock share in 2050 (59% BEVs and 22% stock-equivalent plug-in hybrid electric vehicles [PHEVs]; we count PHEVs as partial ZEVs based on their share of electric driving). However, the most impactful variable on combined light-duty and MHD FCEV adoption is not a ZEV sales mandate, but 100% access to hydrogen refueling. This is primarily because in the baseline, we assume no access to hydrogen refueling stations for LDVs but some access for MHDVs. With 100% of households having access to hydrogen refueling by 2050, FCEV stock reaches nearly 600,000 but remains <1% of the total LDV stock in 2050. No variable in isolation drives significant adoption (>1%) of light-duty FCEVs, suggesting that multiple factors are needed to enable their adoption (i.e., increased refueling access alone is not enough with baseline cost assumptions for hydrogen and FCEVs). In contrast, BEV adoption is increased under several scenarios, demonstrating that individual factors, as modeled here, can support BEV adoption. After mandating 100% ZEV sales, accelerated reductions in vehicle costs (i.e., reduction in battery costs) are the next most impactful variable for achieving high BEV adoption (second row in Fig. 2). Battery costs reaching $40 kWh^−1^ by 2050 in isolation leads to 45% light-duty BEV stock by 2050 and a reduction of 0.20 Gt CO_2_e compared to the 2050 baseline. Convenience of charging is also impactful to accelerating adoption. A lower monetized value of time while charging and increased availability of household charging increases adoption over the baseline (third and fifth rows in Fig. 2). If BEV owners consistently have zero value of time during charging events such as doing something else while “waiting to charge” (e.g., shopping, working), 2050 BEV stock adoption reaches 35%, ceteris paribus. We find for every 2.9% increase in residential charging availability there is 1% increase in 2050 BEV stock (residential charging incurs no time penalty). Changes to fuel prices and fuel economies can moderately impact light-duty BEV adoption in isolation. Carbon prices, increased fossil fuel prices, or decreasing electricity prices can all accelerate light-duty BEV adoption. However, changes to energy prices in isolation are not very impactful on MHD ZEV adoption due to baseline ZEV costs remaining high, such that marginal fuel cost reductions do not sufficiently increase overall ZEV cost competitiveness. Reductions in MHD BEV costs lead to rapid growth in stock share to 41% by 2050 if battery costs drop to $40 kWh^−1^ by 2050. Aggressive FCEV cost reductions (nearly 50% reduction from current) lead to 540,000 MHD FCEVs in 2050. However, when only one variable is changed in isolation, FCEV sales are projected to remain very limited, even under the most optimistic assumptions to individual variables.

These sensitivity results test each variable in isolation against the baseline to evaluate the impact of individual levers on deep decarbonization of passenger and freight mobility. There are many scenarios that show marginal or no impact on mobility emissions, as reductions may be more significant when compounding or synergizing with other polices, behaviors, or technology changes. For example, achieving a significant increase in light-duty BEV fuel economies in isolation does not have much impact on mobility emissions because BEVs remain expensive and lack sufficient charging options in the baseline scenario.

### Uncertainty of U.S. mobility emissions

Here we report results from the multivariate scenario design to explore uncertainty of future mobility emissions and shed light on interactions and synergies across multiple variables. Such multivariate simulations explore a broad array of future scenarios to identify possible pathways towards decarbonization but are not meant to indicate the likelihood of different outcomes.

**Fig. 4:**
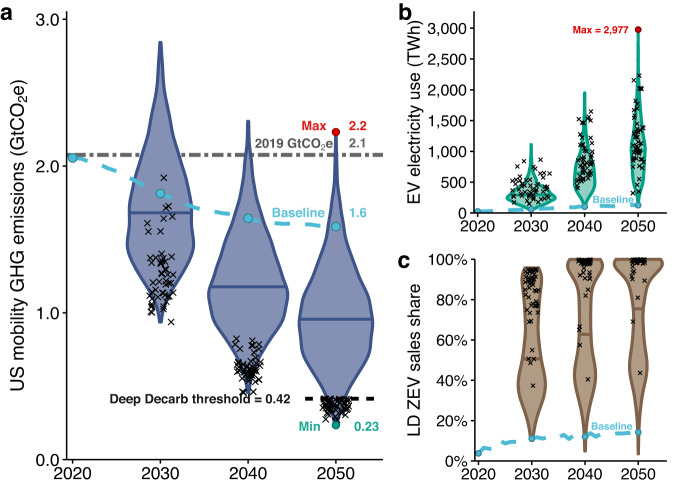
Summary of multivariate uncertainty scenarios. **a** shows variation in modeled outcomes of future decadal U.S. mobility well-to-wheel greenhouse gas (GHG) emissions, (**b**) decadal direct electricity use (electricity for passenger and freight electric vehicles [EVs]), and (**c**) decadal light-duty (LD) zero-emission vehicle (ZEV) sales shares; 2000 scenarios were simulated with 1000 simulations each under a decarbonized and a non-decarbonized power sector. Scenarios that reach deep decarbonization (*N* = 50 in each year) are denoted with black “x” marks in each panel. These results do not indicate the likelihood of outcomes.

Results indicate a large range of outcomes for emissions, electricity use, and ZEV adoption, and most of the scenarios simulated project significant emissions reductions compared to the baseline. Figure 4 shows the spectrum of outcomes for future U.S. transport mobility emissions across 2000 simulated multivariate scenarios (1000 baseline grid and 1000 decarbonized grid). The maximum potential for 2050 decarbonization across the scenarios simulated was an 89% GHG reduction relative to 2019 and an 85% reduction from the 2050 baseline. By 2030, the maximum potential for emissions reductions is 55% from 2019. In this scenario, emissions reductions are driven by 18% lower travel demand, 13% increased vehicle occupancies, 13% sustainable biofuel use in domestic commercial aviation (1.7 billion gallons), 35% light-duty BEV stock (85% sales share), and 13% MHD BEV share (69% sales share), all coupled with a heavily decarbonized electric grid (70% reduced grid emissions from 2019). A wide range of ZEV adoption outcomes (Fig. 4c) combined with uncertain travel demand growth drives a wide range in future electricity demand (Fig. 4b); the median demand for (battery) electricity for mobility is 1000 TWh with a range 120–3000 TWh. In a few scenarios, emissions are projected to increase above the baseline or stay roughly the same and are characterized by high BEV charging costs, reduced system-wide efficiencies (e.g., high-congestion scenarios), and increased total travel demand. This demonstrates the importance of affordable carbon-free electricity and EV charging solutions as well as effective planning for and managing of travel demand to ensure emissions reductions can be achieved.

**Fig. 5:**
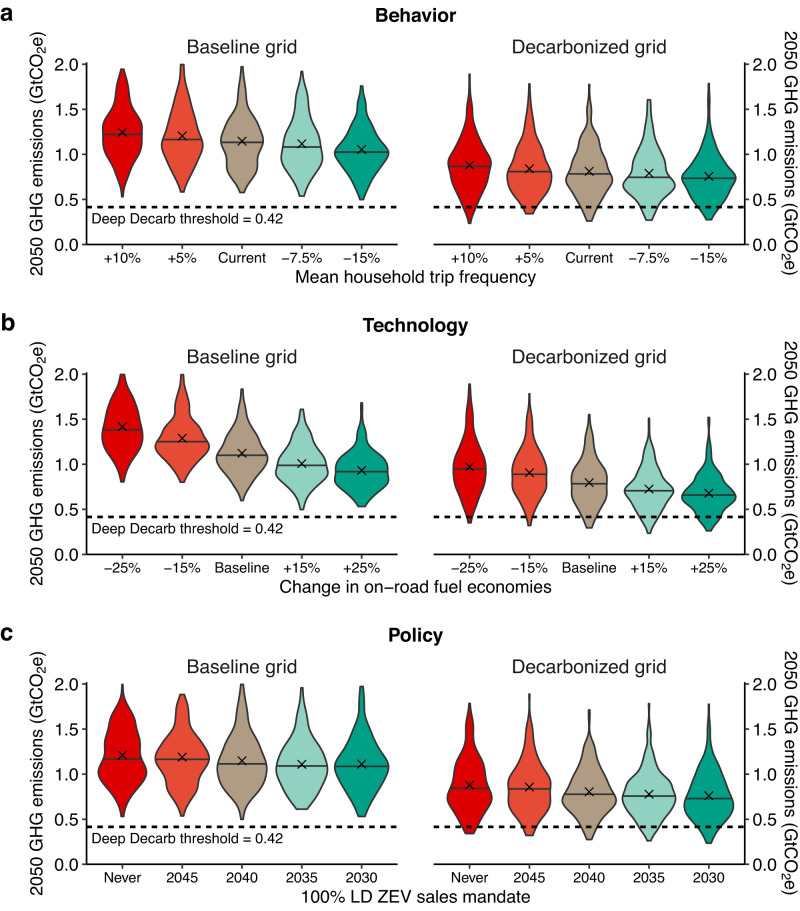
2050 U.S. mobility greenhouse gas (GHG) emissions under a baseline (left) or decarbonized (right) energy grid for three input variables, one of each type: behavior, technology, and policy. Within each input category, we show a commonly discussed lever for transportation decarbonization: **a** demand reduction in passenger trips (behavior); **b** fleetwide on-road vehicle fuel economies (technology); and **c** a light-duty (LD) zero-emissions vehicle (ZEV) sales mandate (policy). Variable means are marked with an “x,” medians with a horizontal line, and the deep decarbonization threshold by a horizontal dashed line. These results do not indicate the likelihood of outcomes.

Pathways to deep decarbonization will require a fully decarbonized electricity grid coupled with transportation changes across dimensions of behavior, technology, and policy. Figure 5 shows the uncertainty ranges in emissions reductions across scenarios under a baseline or decarbonized energy grid for the three most sensitive modeled variables (one each for behavior, technology, and policy). Reducing passenger trip demand (Fig. 5a) is one of the most impactful levers to reduce emissions: A 5% reduction in passenger trip frequency leads to a mean reduction of 0.042 and 0.039 Gt CO_2_e in mobility emissions for baseline and decarbonized grids, respectively. Reducing travel demand is more impactful in scenarios with lower decarbonization, as the emissions intensity of travel is higher. Improving on-road driving efficiencies (one of the most impactful technological categories, Fig. 5b) also has stronger impacts on emissions reductions in scenarios with lower decarbonization; improving all-duty on-road fuel economies by 5% leads to a mean reduction of 0.064 and 0.032 Gt CO_2_e in mobility emissions for baseline and decarbonized grids, respectively. While ZEV mandates were highly impactful in isolation, the direct impact to emissions reductions across uncertainty runs is less significant because of many other variables influencing competitiveness and viability of ZEVs. This indicates that a ZEV adoption mandate might not be essential if other factors supporting ZEVs (especially technological) are realized. No uncertainty scenarios reach deep decarbonization without a decarbonized grid (no scenarios occur below threshold in left pane of Fig. 5), and the mean difference between scenarios under baseline versus decarbonized grid is 0.34 Gt CO_2_e (21% reduction from baseline 2050 emissions). These results underscore the importance of power sector decarbonization to reach the full potential to mitigate transportation emissions.

The adoption of ZEVs has a large impact on emissions reductions, and results show a large range of outcomes in both the passenger light-duty and freight MHD sectors. Supplementary Fig. 4 shows the range of outcomes for ZEV stock and sales shares. For LDVs, ZEV stock varies from 7.8% to 83% in 2050, while MHD ZEVs make up 0% to 88% of the vehicle stock in 2050. The most consistent assumption in scenarios with high ZEV success (light-duty or MHD shares over 80% by 2050) is a higher differential in total cost of driving (i.e., pessimistic conventional vehicle costs and/or optimistic reductions in ZEV costs). Energy prices are less impactful but still significant for ZEV adoption. A carbon price of $500 per tonne CO_2_e has a mean impact of +11% light-duty ZEV stock share by 2050. ZEV adoption also benefits from increased availability of charging and refueling infrastructure. In the baseline, we assume that 11% of households have access to residential EV charging^34^, and the most aggressive availability of home charging increases to 75%, increasing the 2050 BEV stock share by a mean of 11%. For light-duty FCEVs, 100% household access to hydrogen refueling leads to a mean of 10% light-duty FCEV stock share (mean 32% sales share) in 2050 when combined with baseline assumptions for BEVs and at least one of the following: lower consumer hydrogen prices (as low $3.40 kg^−1^ with $1 kg^−1^ for production), lower FCEV costs, or higher FCEV fuel economies. Note that this level of adoption might not justify full refueling infrastructure deployment. For MHD vehicles, 100% fleet access to hydrogen refueling leads to a mean of 36% FCEV stock share in 2050 (mean 55% sales share) when also assuming baseline assumptions for BEVs combined with at least one of the following: lower hydrogen prices, lower FCEV costs, or higher FCEV fuel economies.

Biofuel use across scenarios can also vary significantly with use in on-road and aviation applications depending on policy and travel demand evolution. In 2019, the United States consumed 16 billion gallons of biofuel^35^. We project by 2050 total mobility-related domestic biofuel consumption could range between 1.5 and 61 billion gallons (0.12–7.8 EJ), with emissions from biofuel of −0.092–0.25 Gt CO_2_e depending on the evolution of biofuel production pathways and the assumed well-to-wheel GHGs for biojet and biodiesel. The most extreme scenario of biofuel use (without limiting supply) is dominated by biojet fuel: 47 of the 61 billion gallons consumed in 2050 are biojet fuel due to increased competitiveness of aviation from reduced rail availability and significantly increased fossil fuel prices. However, most scenarios use less biojet fuel even when it fully replaces conventional jet fuel, with a median of 8.7 billion gallons biojet fuel use per year by 2050 and a median of 51% of aviation demand supplied by biofuels (considering domestic travel only; these number could increase significantly when considering international travel). The uncertainty and rate of ZEV adoption is the driving factor for the rest of biofuel use; on-road biofuel use peaks in the 2030s with a max of 33 billion and a mean of 12 billion gallons, and by 2050 mean on-road use is 8 billion gallons. Supplementary Fig. 5 summarizes the range of electricity, hydrogen, and biofuel demand over time for all scenarios.

### Pathways to deep decarbonization

**Fig. 6:**
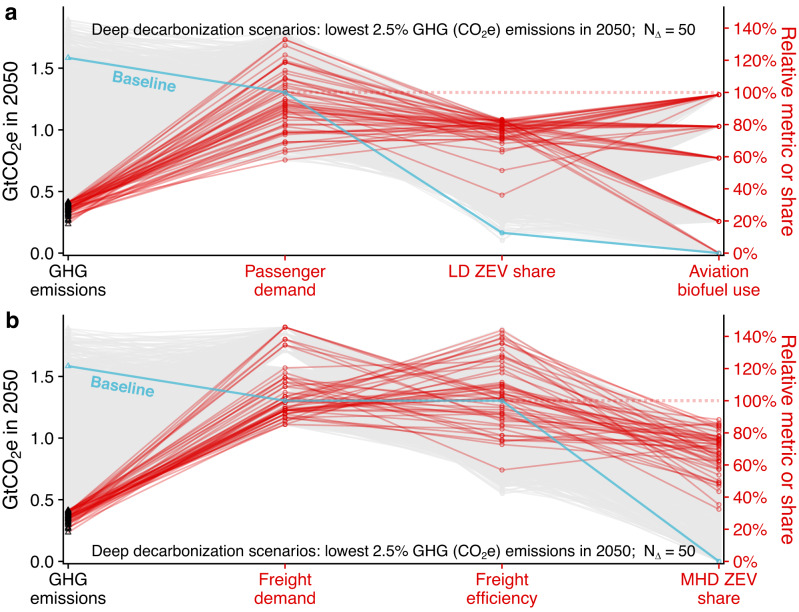
2050 deep decarbonization pathways connecting passenger and freight mobility greenhouse gas (GHG) emissions. Emissions for passenger (**a**) and freight (**b**) travel are shown as black triangles (left) and correspond to the left axes. Pathways to key outcomes are shown in red and correspond to relative values or vehicle market shares scaled to the right axes. These highlighted scenarios are the 50 lowest-emission scenarios in 2050 (2.5% lowest 2050 emissions out of 2000 total scenarios). Demand is measured in relative units to the baseline, with 100% indexed to 2050 demand of 6.6 trillion passenger-miles traveled (**a**) and 5.0 trillion tonne-miles traveled (**b**). Freight efficiency is also measured in relative units to the baseline, with 100% indexed to 148 tonne-miles per gasoline gallon equivalent (GGE).

To understand what combinations of factors can contribute to deep transportation decarbonization, we evaluate the lowest-emissions scenarios more closely in this section. We define ‘deep decarbonization’ as an 80% reduction in transportation mobility well-to-wheel GHGs from the 2050 baseline; this corresponds to the 50 lowest-emissions scenarios out of 2000 total scenarios simulated (0.42 Gt CO_2_e in 2050 or less). These 50 scenarios have mean emissions of 0.27 Gt CO_2_e in 2050 compared to the 2050 baseline of 1.6 Gt CO_2_e (83% reduction) and a reduction of 87% from 2019 emissions. Figure 6 shows pathways to deep decarbonization across key variables for passenger and freight travel. All deep decarbonization scenarios fall in the fully decarbonized electric grid case, which reduces well-to-whell GHGs to zero by 2035 for BEVs, FCEVs (all fueled with electrolytic hydrogen), and PHEVs while driving on battery-electric power. While the feedbacks between the power and transportation sectors are not considered here, multiple studies have shown that large-scale transportation electrification can also help power system decarbonization and provide demand-side flexibility^36,37^, further emphasizing the synergies between these two sectors in achieving deep decarbonization^38^.

In these scenarios, an important driver of deep decarbonization is reduction of travel demand: 88% (44 of 50) of deep decarbonization scenarios have reductions in passenger or freight travel demand, and 46% of scenarios have reductions in both passenger and freight demand. Eighty percent of deep decarbonization scenarios have reductions in passenger travel demand (reductions of household trip frequency, trip length, or both), with 50% having at least reductions in passenger trip frequency and 34% having reductions in both passenger trip frequency and trip length. Twenty-four percent of scenarios assume the highest reduction to frequency (15% fewer trips). Reductions in freight demand are also prevalent, with 54% of deep decarbonization scenarios having reductions in freight demand (reductions of tonne-miles demanded, freight trip lengths, or both). However, only 12% of deep decarbonization scenarios have both types of freight demand reduction. For details on modeling assumptions for changes to travel demand, see Supplementary Section 3.

ZEV adoption is a significant contributor to achieving deep decarbonization by 2050. Deep decarbonization scenarios have a mean of 76% light-duty stock share (96% sales share) and a mean of 68% MHD stock share (97% sales share) in 2050. To reach deep decarbonization by 2050, these scenarios have a mean of 82% light-duty sales share and 63% MHD sales share in 2035. Increased demand for mobility makes decarbonization somewhat more difficult with increased ZEV needs in 2050; scenarios without reductions in baseline passenger demand have a mean of 78% LDV stock share (98% sales share), and scenarios without reductions in baseline freight demand have a mean of 78% heavy-duty vehicle stock share (100% sales share). In the freight sector, only 16% of scenarios reach >80% ZEV stock by 2050, usually characterized by BEV dominance; however, 22% of deep decarbonization scenarios show a greater share of MHD FCEVs over BEVs. Crucial to the success of FCEVs is high access to refueling infrastructure, as well as low price of clean hydrogen to consumers ($6 kg^−1^ or lower by 2040) and major improvements in FCEV costs and performance. On top of success for hydrogen technologies, scenarios showing major MHD FCEV adoption also have increases in shipment lengths and pessimistic assumptions for BEVs (higher electricity prices, more expensive batteries, and 2-year freight vehicle payback requirement for truck adoption). There was one deep decarbonization scenario with low light-duty ZEV adoption by 2050 (Fig. 6a); 36% of ZEV stock was composed of 3.0% BEVs, no FCEVs, and 75% PHEVs with 52% of light-duty miles driven on electricity in 2050. Decarbonization is achieved in this scenario due to 100% use of sustainable biofuel in domestic aviation (13 billion gallons), decreases in passenger and freight travel demand, 72% MHD ZEV share, and increased vehicle occupancies. The favor for hybrids in this scenario is driven by improved PHEV costs and fuel economies relative to other technologies. In these simulations, we treat technology inputs for different applications such as light- and medium-/heavy-duty vehicles independently, so different levels of success in different subsectors are possible in some scenarios for a technology in each subsector. While most scenarios include some mandate for ZEV sales, 24% (12 of 50) do not have any type of mandate in at least one subsector; five with no light-duty ZEV sales mandate, eight with no MHD ZEV sales mandate, and one with no ZEV sales mandates at all. ZEV success in these scenarios derives from greatly improved ZEV cost competitiveness.

Deep decarbonization pathways without reduced passenger or freight travel demand show higher ZEV market shares and higher transportation-related electricity needs. Scenarios with decreased passenger and freight demand result in an average of 1000 TWh of direct transportation electricity use in 2050, while scenarios with increased passenger and freight demand require an average of 1800 TWh in 2050 and can reach as high as 3000 TWh (Fig. 4b). Direct transportation electricity use only includes electricity consumed by BEVs and electrified rail. High amounts of indirect electricity may also be needed in deep decarbonization pathways to support hydrogen production for use in vehicles, reaching as high as 3100 TWh in 2050 assuming all hydrogen is produced using electrolysis at 51.3 kWh per kg H_2_ (this hydrogen demand is nearly equivalent to the current global demand of hydrogen^39^). Scenarios with decreased passenger and freight demand result in an average of 320 TWh of indirect electricity for hydrogen production in 2050, while scenarios with increased passenger and freight demand require an average of 750 TWh. When combining direct and indirect electricity demand, high ZEV adoption could necessitate significant expansions of decarbonized electricity-generating capacity; scenarios without reductions in travel demand require additional electricity supply between 1100 and 4300 TWh in 2050 to achieve deeply decarbonized mobility in the United States (but under 2200 TWh for scenarios with reduced passenger and freight travel demand). The upper limit to total electricity demand is the most extreme scenario of FCEV adoption, with 2050 light-duty stock of 32% (72% sales share) and MHD stock of 67% (100% sales share), resulting in 60 Tg H_2_ consumed in 2050. Additional electricity might also be needed to support biofuel (via hydrogen) of synthetic e-fuel production, but it is not considered here.

Increased use and further well-to-wheel emissions reductions of sustainable biofuel is essential for deep decarbonization, especially by reducing the carbon footprint of aviation travel and supporting reduced emissions while transitioning away from fossil fuels (legacy vehicles). Exceptions to high biofuel penetrations in deep decarbonization scenarios are due to demand reductions, especially in aviation. Over one-third (36%) of the deep decarbonization scenarios utilize 100% biojet fuel use for (domestic) aviation, while only three (6%) scenarios have no biojet fuel use. These scenarios reach deep decarbonization without biojet fuel use because of (1) high biofuel use in other modes with 50% reduction in well-to-wheel biofuel emissions, (2) reduction in passenger demand, and (3) additional shifts away from longer-distance air travel due to the combination of increased fossil fuel prices and increased availability of commuter rail. On-road biofuel use stays stable or decreases relative to current total transport biofuel use; this is driven by ZEV adoption demand growth rates with a maximum of 18 billion gallons consumed for on-road applications in the 2030s and all decarbonization scenarios under 8 billion gallons by 2050.

## Discussion

This paper takes a novel approach of simulating thousands of transportation futures under a wide range of plausible input assumptions to provide insights of pathways to decarbonize U.S. passenger and freight mobility. The future is unpredictable, and the purpose of this study is not to predict the likelihood of future outcomes. Instead, we evaluate various pathways that reach deep decarbonization of U.S. mobility to guide future decision-making and energy system planning. Many pathways exist to decarbonizing travel, but these results show that there is no single silver bullet; instead, multiple levers need to be pulled to achieve deep decarbonization of U.S. mobility, and we highlight the implications and trade-offs of various pathways in the transformation of the transportation energy system. We consistently find that a rapid and widespread transition to ZEVs is a key requirement of decarbonization for on-road passenger and freight mobility, and we quantify the range of future electricity expansion needed to support a decarbonized mobility.

We find no significant shifts away from personal cars (±5%), even under scenarios with shortened trip lengths and increased transit access. Past research has highlighted a strong consumer preference for personal car ownership and use due to the flexibility and time-efficient access they offer^23^, structural barriers hindering other options^40,41^, underestimating their costs^42^, and other cultural factors^43–45^. Many other studies project no significant change to personal car use and similarly see ZEV adoption as key driver of transportation decarbonization^5,10,46,47^. However, some transportation decarbonization pathways assume much greater shifts away from personal cars: IEA’s “Net-Zero by 2050” scenario^8^ sees 20–50% of global urban car trips shifting to active and public transit by 2050, and other studies highlight the need for mode shifting and demand reduction to supplement electrification to meet decarbonization targets^18–22^.

Besides vehicle electrification, reducing overall travel demand is shown to be the most consistent driver of emissions reductions because it avoids emissions entirely, but it remains one of the hardest strategies to implement. Future travel needs are highly uncertain but expected to grow with increasing population and economic activity^48^, yet we find 88% of pathways reaching deep decarbonization have reductions in passenger or freight mobility. Reduced demand (fewer or shorter trips) can result from a combination of better urban planning, travel demand management, digitalization (e.g., telework), and better incentives to choose sustainable travel options. However, large reductions in travel demand are less frequently considered in technology-focused decarbonization strategies given the major behavioral and cross-sectoral planning changes required, as well as the need to maintain the economic and social benefits of travel (e.g., access to goods, services, jobs, and other people is essential to a high-quality life^49^). One study found that while the frequency of trips does not impact quality of life, unserved travel demand does negatively and strongly^50^. In the wake of the COVID-19 global pandemic, rising remote activity engagement (e.g., telework, e-learning, e-shopping, telehealth) occured^51^, unlocking unforeseen opportunities to avoid emissions and physical travel in favor of virtual access^52–55^ but total travel demand has largely rebounded to pre-pandemic levels^56^. Another opportunity to reduce travel demand is by reducing the overall proximity of destinations (e.g., shorter trips via changing urban form)^57,58^. Literature consistently finds that more sprawling development increases per-capita energy consumed for travel^59–64^, indicating that changes to urban form could lead to lower emissions footprints and easier-to-manage energy needs. Not only does better proximity to opportunities decrease travel, but it also increases the competitiveness of other sustainable travel options such as walking or micromobility, which can further reduce emissions^65–67^. These options emit significantly less GHGs per passenger-mile over their life cycle compared to private cars^68^, but in many U.S. cities, sprawled development and a lack of quality infrastructure and complementary public transit further undermines their adoption and limit the effectiveness of these strategies in reducing future emissions.

Our results show that deep transportation decarbonization can be achieved even without travel demand reductions under several plausible assumptions, but still requires a rapid and massive transition to ZEVs that outpace most previous estimates. Scenarios achieving deep decarbonization without major reductions in passenger and freight demand reach a median of 89% light-duty ZEV sales and 69% MHD ZEV sales by 2030, and by 2040, the median scenario has both segments reaching 100% ZEV sales. Projections tied to reaching decarbonization goals show similar but sometimes less extreme electrification. Hitting targets of technology advancement may bring MHD ZEV price parity with conventional technologies by 2035 but 100% sales shares would still not be achieved until at least a decade later^69^, and other studies project similar or slower adoption in the MHD sector^11,70^. Alarfaj et al.^22^ find similar light-duty trends with 2040 being the last year for full ZEV market share to ensure meeting 80% decarbonization targets. One of the most aggressive adoption scenarios for light-duty, 70% EV sales in 2030 and 100% sales by 2040 occurs alongside an 80% increase in travel demand^20^. However, many other studies find achieving net-zero economy-wide emission with slower adoption rates^10,19,21,46^.

Widespread use of ZEVs will be a key strategy for transportation decarbonization, but with increasing travel demand, they will lead to massive increases in electricity demand in the transport sector. Previous studies find between 1100–2000 TWh of additional direct (battery) electricity demand could be needed by 2050 to support high electrification in the United States under different assumptions with various limitations. High vehicle electrification leads to 1500 TWh in Mai et al.^11^ and up to 1100 in Ou et al.^46^ by 2050, but both studies assume no adoption of FCEVs and steady travel demand growth. Larson et al.^10^ find 1900 TWh is needed for directly electrified transport by 2050 in their high electrification with 100% renewable energy scenario, and indirect electricity needs from electrolyzed hydrogen for FCEVs reach ~800 TWh. Milovanoff et al.^20^ evaluated three future pathways of electrification to meet targets and found 1100–2000 TWh of additional energy demanded in 2050 but only for light-duty EVs under travel demand increases of 40-80%. Our sector-wide results provide similar trends but with higher ceilings of future transportation electricity needs due to additional uncertainties. The median deep decarbonization scenario without reductions in travel demand results in 1500 TWh of direct (EV) electricity demanded by 2050, but future travel growth causes uncertainty between 1100–3000 TWh (high end is three quarters the size of the current power sector). In addition, electricity demand for electrolyzed hydrogen production for transportation applications ranges from zero to up to 3100 TWh by 2050 (without considering hydrogen for biofuel processing or for use in other sectors). When considering direct (battery) electricity plus indirect (electrolyzed) hydrogen needs, some scenarios see total transport electricity demands over 4000 TWh in 2050 due to significant adoption of FCEVs (primarily in freight). Higher reliance on hydrogen or synthetic fuels greatly exacerbates electricity demand growth compared to BEVs but might be the only viable solution for some applications. These are major increases to current electricity generation of 4000 TWh^71,72^. While we did not evaluate hourly and seasonal peak demand, this further highlights the need to consider managed EV charging approaches especially if it can synergize with renewable generation^38^. Still, some uncertainty remains regarding other indirect demand in the sector (e.g., to support increased biomass production, vehicle manufacturing) and other possible contributors not evaluated such as electrified aviation and off-road vehicles. Travel demand growth coupled with widespread ZEV adoption will cause far-reaching implications requiring timely and careful planning to ensure adequate decarbonized electricity supply and further highlights the value of travel demand management to ease future needs for constrained low-carbon electricity supplies.

Increased use and reduced GHG emissions of biofuels also play a critical role in transportation decarbonization, as complete shifts away from fossil fuels may be very difficult for some modes (e.g., aviation) and are unlikely in the near term, thus leaving some legacy vehicles operating on liquid fuels in 2050. Sustainable biofuel use can also help offset increased travel demand during transitions to ZEVs. Moderate to high use of aviation biofuel is common in deep decarbonization scenarios, and without reduced demand for long-distance travel in the United States, we estimate 10–42 billion gallons yr^−1^ (1.3–5.5 EJ) of biojet fuel needed by 2050 to ensure deep decarbonization of domestic passenger travel. This results in 14–152% decrease in 2050 aviation emissions relative to 2019 (0.16 Gt CO_2_e) with uncertainty driven by the magnitude of well-to-wheel emissions reductions in biojet production, growth in travel demand, and the level of decarbonization in non-aviation mobility. Demand for sustainable aviation fuel could also approximately double when considering international travel and military needs. Maritime biofuel will also be important in decarbonization strategies, but this study focused on domestic freight, which includes marginal maritime energy use. Projections for biomass availability in the United States indicate that most scenarios modeled here could fit within sustainable supply projections: if multiple production pathways are successful, the United States could produce 50–60 billion gallons of sustainable low-carbon biofuels per year^73^. However, these biomass supply constraints may impact the level of transportation decarbonization achievable given competition for consumption as aviation may need a large allocation of future supply. Regardless of supply constraints, improvement in biofuel life-cycle emissions of 50% or more compared to fossil fuels are essential for biofuels to play a large role in deep decarbonization of transportation.

Multiple uncertainties remain about the evolution of future mobility systems, and this study does not capture every possible element impacting a transition to a sustainable future. Travel demand reduction impacts are assumed exogenously for some scenarios, and we do not consider certain endogenous feedbacks to travel demand that could occur, such as passenger demand changes impacting freight demand or vice versa (e.g., increased e-commerce increasing freight demand but reducing passenger demand); the change in travel demand due to changing costs (e.g., carbon pricing could curtail long-distance recreational or leisure travel; or changes in demand due to changes in urbanization or migration). In addition to impacting total aggregate demand, future shifts in urbanization may impact mobility needs and preferences due to underlying changes in the distribution of trip needs. While we do not directly model urbanization impacting demand endogenously, we exogenously vary trip length and trip frequency distributions to capture expected urbanization continuing in the United States^74^.

We focus on well-to-wheel GHG emissions but exclude impacts from vehicle manufacturing, as these are smaller and closely linked to industrial decarbonization. Also, we do not consider emissions from the life cycle of necessary infrastructure that supports travel. Previous research has noted the high pollution from various transportation infrastructure, especially concrete production and vehicle production^75^, so pathways that reduce travel demand would also benefit from additional life cycle decarbonization. While we focus on GHG emissions, criteria air pollutants—which are important for local policy to improve air quality—can also be mitigated by ZEVs.

Some modes of travel are not evaluated due to limitations of data and our modeling approach. Due to limited observational data at the national level, we do not consider autonomous vehicles (AVs) or electrified micromobility (e-bikes and e-scooters), and we do not separately consider biking from walking, including the increase in speed from biking. While we did not explicitly model AVs, some of the modeled variables independently and in combination could resemble futures with more automated travel. For example, the benefit of increased on-road fuel efficiencies is one common improvement from vehicle automation^76^, and the potential for increased travel demand is a potential drawback to (especially private) AV adoption by increasing urban sprawl and decreasing value of travel time^77^. While fewer deep decarbonization pathways exist with higher passenger travel demand, AVs may compete with medium- and long-distance travel^78^ and are likely easier to decarbonize than aviation. As we did not include micromobility, we did not evaluate its potential to replace car trips under shorter trip length scenarios. Previous research has shown emissions reductions are possible from mode shifts to micromobility, especially in more urban areas where shorter trips are most frequent^79^. Shifting shorter trips to micromobility and enabling car trip replacement could act as a substitution to demand reduction scenarios we evaluated (reducing trip frequency shifts more trips to shorter distances and fewer trips to longer distances, thus decreasing overall demand; see Supplementary Section 3 for details).

It is important to note that TEMPO does not currently compete biofuel options nor limits total supply (i.e., we do not integrate with a supply-side model for biomass production). In addition, aviation technologies are limited to decarbonization with biojet fuel only to some degree. As a result, this study assumes that biomass production may be heavily targeted towards aviation and doesn't consider economy-wide constraints that may otherwise limit the extent of decarbonizing air travel.

This study highlights some opportunities for future research and to improve the representation of mobility in decarbonization analyses. We evaluated some scenarios that impact Mobility-as-a-Service (MaaS; e.g., taxis, on-demand services, and other ridesharing) but did not find any significant impacts from changing the cost or time to users. Other studies, however, have pointed to an increased competitiveness of MaaS potentially reducing household vehicle ownership that should be further studied^80–82^. Given the high impact on emissions of assumed demand changes, capturing endogenous demand shifting and projections of urbanization would be valuable to understand other possible future pathways to emissions reductions. Decarbonizing long-distance travel will be critical to achieve deep decarbonization, but TEMPO currently has limited alternatives to and technology options within aviation, and we do not assess potential for long-distance trips in AVs nor explicitly explore scenarios of targeted and high build-out of passenger rail systems (e.g., significant increases of intercity and high-speed rail). Future research should consider this an important topic as it may be critical to facilitate decarbonizing long-distance travel. Better representation and study of EV charging behaviors may also further elucidate the role of infrastructure built-out on EV adoption and resulting decarbonization. There are still gaps in understanding how investments in public alternatives for recharging and refueling infrastructure could contribute to reduced emissions by supporting ZEV adoption, especially among households with lower income and no access to private home charging.

Direction is urgently needed to inform transportation and energy systems planning and investments to achieve ambitious emissions reductions. We explore future pathways of U.S. mobility-related well-to-wheel GHG emissions leveraging thousands of scenarios under different future assumptions about mobility behavior, technology characteristics, and policies. This study demonstrates that many factors such as the speed of ZEV adoption and the rate of travel demand growth will impact deep decarbonization of U.S. passenger and freight mobility. Results show that pathways to deep decarbonization all include widespread adoption of ZEVs in both passenger and freight sectors supported by a decarbonized electric grid. This is achieved primarily through high access to refueling options, cheaper clean energy prices relative to fossil, and lower ZEV prices. Most decarbonization pathways are dominated by EVs especially in light-duty, however FCEVs gain more success in MHDV with a fifth of scenarios having greater FCEV adoption over BEVs. This high ZEV use necessitates significant growth in electricity demand with major trade-offs determined by the share of BEVs and FCEVs. These findings shed light on the high uncertainty of future decarbonized transport-electricity needs and stress the importance of managing travel demand to ease these needs for limited low-carbon electricity supply and optimize the build-out and use of the electricity system. Overall, the transformations needed to decarbonize mobility will play a key role in the long-term evolution of the power sector, calling for highly integrated analysis and planning. These paths will be critical to advance any decarbonization strategy both to understand the impacts and implications of these various pathways and inform decisions and trade-offs to achieve deep emissions reductions in mobility.

## Methods

### TEMPO model overview

The TEMPO model was developed to provide a framework to investigate long-term scenarios of mobility energy use and emissions^32^. Distinct capabilities of the model relevant to this analysis include representation of household-level travel decisions for travel demand, vehicle ownership, mode choice, and technology adoption for various geographies (e.g., urban, suburban, rural) and sociodemographic groups (e.g., income, household composition), and disaggregation of freight travel demand by operating segments. This segmentation allows for exploration of various influences on mobility evolution including technology advancement and changes in demand, and it enables a robust estimation of technology adoption potential and energy use impacts across market segments. Using TEMPO, we model the entire U.S. domestic passenger and freight mobility system across all major travel modes including active mobility (walking and biking), light-duty personal travel, MaaS, public and intercity transit, domestic aviation, domestic (maritime) ship freight, passenger and freight rail, and freight trucking. For this study, TEMPO is used to generate endogenous scenarios of mode choice and technology adoption for passenger and freight mobility based on factors like vehicle cost and fuel economy, fuel cost, availability of refueling/charging options, varying travel needs, availability of alternative modes like transit, and various policies.

A baseline TEMPO scenario was developed that uses consistent assumptions with and closely matches energy use by fuel, mode, and technology in the U.S. Energy Information Administration (EIA) AEO 2019 Reference Scenario^83^. The 2019 AEO Reference was chosen for several reasons: (1) to have a consistent initial year with TEMPO sourced demand data from the 2017 National Household Travel Survey^84^ and Freight Analysis Framework^85^; (2) to match a scenario that assumes no significant progress in technology, behavior, or policy; and (3) to avoid a scenario that is impacted by significant short-term changes from COVID (long-term changes that may result from COIVD are still considered). The baseline scenario offers a baseline for comparison of other scenarios and illustrates the ability of TEMPO to comparably represent the key elements (e.g., mode share, energy use) that determine the evolution of transportation systems over time. This TEMPO scenario is used in this study as the “baseline” to serve as a reference point of comparison for sensitivity and uncertainty analyses to ensure our reference point for comparisons assumes no significant progress in technology, behavior, or policy (e.g., 13% vehicle electrification in 2050 and continued petroleum dominance). An overview of this TEMPO baseline comparison to AEO is displayed in Supplementary Section 2. For a more comprehensive overview of the TEMPO methodology and validation, see Muratori et al.^32^.

### Study design and inputs

To address uncertainty and inform how deep decarbonization could be achieved, we conducted 2173 TEMPO simulations to 2050, varying multiple inputs spanning assumptions on the evolution of technology costs and performance, consumer travel behavior, and policies based on targeted expert elicitation. This novel study design is intended to span optimistic and pessimistic bounds to capture a wide space of plausible futures rather than identifying “most likely” input assumptions and carefully designed scenarios to avoid bias or missing unforeseen synergies. We do not assign any probability or likelihood to any inputs or outcomes. Instead, this study design is focused on exploring uncertainty and evaluating feasible ranges of passenger and freight travel emissions under a broad array of future scenarios (e.g., possibilistic but not probabilistic) based on a wide set of bounded inputs.

We perform two exercises: (1) change a single input variable at a time to quantify and rank the isolated impact (sensitivity) of each input variable on results; and (2) change multiple input variables in parallel to explore the broadest landscape of possible futures (uncertainty). The univariate sensitivity scenario design focuses on ranking inputs on their ability to impact emissions (or other outcomes) in U.S. passenger and freight travel in isolation against the baseline. The multivariate scenario design focuses on mapping how combinations of variables affect the evolution of emissions and enable discovery of pathways to deep decarbonization.

We evaluate sensitivity and uncertainty by varying 51 exogenous input variables over 222 distinct assumptions. For each input variable, we leverage existing literature and interviewed subject matter experts to identify bounds to represent extreme yet possible potential futures (pessimistic and optimistic) with intermediate points. In some cases, intermediate assumptions are spaced evenly between the bounds, while for other inputs the intermediate values correspond to existing projections or assumptions from policy targets. We focus on using trajectories to model how input variables change over time. The trajectories vary such that the longer-term (i.e., 2050) values exhibit more uncertainty than the near-term values. For example, fossil fuel prices vary by 50% from AEO 2050 values; however, the deviation from the AEO trajectory is interpolated from 0% in 2020 (no uncertainty in historical fuel prices) to 50% in 2050. A few inputs are not time-dependent within the model (e.g., LDV retirement rates, payback periods for freight trucks), and thus the sensitivity was applied to all years in a scenario, but we focus most on impacts in 2050. TEMPO treats each of these exogenous inputs as independent of each other (e.g., changes to refueling availability do not directly impact fuel prices), but mode and technology choice are endogenously captured by different evolutions of the availability, cost, and time of travel options. For more details on the endogenous behaviors and feedbacks captured in the TEMPO model, see Muratori et al.^32^.

Input variables selected for the analysis include assumptions on vehicle technologies (e.g., vehicle costs and fuel economies), fuels and fueling infrastructure (e.g., fuel and electricity prices, refueling/charging availability), household behavior (e.g., vehicle ownership, travel demand), system-level characteristics (e.g., transit availability, travel efficiency), and policy (e.g., ZEV sales mandates, carbon pricing). While TEMPO includes many other input variables, we focused on variables affected by major uncertainty and that may significantly impact future energy use and emissions. For a complete list of input variables considered in this study, see Supplementary Table 1. Inputs about household behavior and mobility demand are meant to capture aggregate effects of better urban planning, greater availability of services close to where people live, effects of digitalization (e.g., telework), and better access to and use of active (biking and walking) travel modes, but we do not model active modes individually.

When combining multiple variables in uncertainty simulations, the combination of variables is created using a quasi-uniform sampling approach. Only one input variable from each of the 51 categories can be modeled simultaneously. Even so, the total possible combinations of simulations that could be run in this variable space is >40 × 10^30^. We use a Sobol sequence algorithm^86^ to choose quasi-random, low-discrepancy sequences and sample a quasi-uniform distribution of variable combinations. We limit to a computationally feasible sample for the multivariate uncertainty analysis, which resulted in 2000 simulations of the TEMPO model on the National Renewable Energy Laboratory’s high-performance computer Eagle, producing over 4 TB of raw simulated output data. The univariate (sensitivity) simulations model only one input at a time over the baseline value, resulting in an additional 173 simulations for a total of 2173.

### Emissions scope (system boundaries)

TEMPO models domestic passenger and freight travel in the United States and endogenously accounts for direct tank-to-wheel energy consumption and emissions (i.e., tailpipe/use phase) from all passenger and freight mobility. We exclude other segments that do not serve passenger or freight mobility including off-road vehicles, recreational boats, military vehicles, lubricants, and pipelines. Excluding these categories, the U.S. Environmental Protection Agency (EPA) reports domestic passenger and freight mobility were responsible for 1.82 Gt CO_2_e tank-to-wheel GHG emissions in 2019 (including all categories and international travel, the sector directly emitted 2.31 Gt CO_2_e)^33^. While TEMPO focuses on modeling the use-phase of transportation systems, other life cycle impacts can be tracked (e.g., emissions associated with producing vehicles or producing the fuels used on-board vehicles) by coupling TEMPO with supply-side models or by using emissions factors from life cycle assessments. To better assess decarbonization potential, we complement TEMPO direct transportation emissions with indirect emissions from energy production by using well-to-tank emissions factors from the Greenhouse Gases, Regulated Emissions, and Energy use in Transportation (GREET) model^87^. As such, we focus on well-to-wheel GHG emissions but exclude other life cycle emissions associated with vehicle production and transportation infrastructure. As a result, we estimate 2.08 Gt CO_2_e were emitted in 2019 to support domestic passenger and freight mobility. We do not model any criteria air pollutant emissions (e.g., from tires, brake pads, or combustion of fuels) or other climate and environmental impacts directly or indirectly associated with transportation (e.g., contrails, land use change associated with biomass production). Scenarios that implement carbon pricing consider well-to-wheel CO_2_e emissions. We treat the decarbonization of the power sector independently with two bounding alternatives for future well-to-wheel emissions from electricity generation based on annual average grid emissions intensities (all multivariate scenario combinations are run under two evolutions of grids): a baseline grid that follows the “Standard Scenario Mid-case” mix^88^ resulting in 210 gCO_2_e kWh^−1^ in 2050, and a case in which the power sector reaches net-zero GHG emissions by 2035 (thus excluding any additional upstream emissions).

The scope of this study focuses on U.S. mobility (passenger and freight movements) within the transportation sector but excludes travel for vocational and service purposes or other non-mobility use within the sector. We exclude service vehicles (utility and emergency), off-road vehicles (construction vehicles, agriculture equipment, and off-road recreation vehicles), commercial light trucks, recreational boating, military travel, pipelines, lubricants for vehicles, and international travel. Based on AEO 2019 sector categories, these excluded categories account for 15% of transportation sector energy use^32,83^. We also do not explicitly model the following technologies: electrified aviation, micromobility modes (e.g., e-bikes), AVs, human-powered bikes and scooters, or high-speed rail. Passenger mobility includes the mode of “no energy,” which assumes walking speeds, and we do not classify other similar zero-energy non-vehicle modes like bicycling. TEMPO currently does not consider ZEVs for non-road sectors except for passenger rail.

While the focus of this study is on emissions outcomes, we often discuss outcomes of stock shares of ZEVs as they are key technologies to decarbonize on-road travel. We consider BEVs and FCEVs as full ZEVs, and we count PHEVs as partial ZEVs according to the mileage driven on electricity (e.g., we assume that a 50-mile-range PHEV drives 62% of its yearly miles on electricity and count it as 0.62 of a ZEV and otherwise operates in charge sustaining mode).

### Supplementary information

Supplementary Information

Peer Review File

## Data Availability

Data for this study are available from the corresponding author on request.

## Code availability

This study leveraged the National Renewable Energy Laboratory’s Transportation Energy & Mobility Pathways^TM^ (TEMPO) model. The model has been peer-viewed and is documented, including a validation, in M. Muratori et al., “Exploring the Future Energy-Mobility Nexus: The Transportation Energy & Mobility Pathway Options (TEMPO) Model,” *Transp. Res. Part D Transp. Environ*. 98, 102967 (2021). More details and code access are available from the corresponding author on request.
