# Supplementary material for: Exploring decarbonization pathways for USA passenger and freight mobility
Source: Nat Commun. 2023 Oct 30;14:6913. doi: 10.1038/s41467-023-42483-0 (PMC10616282; doi:10.1038/s41467-023-42483-0)
Supplement: Supplementary file 1 — Supplementary Information [file 41467_2023_42483_MOESM1_ESM.pdf]

Exploring decarbonization pathways for U.S. passenger and freight mobility

Christopher Hoehne[1], Matteo Muratori[1*], Paige Jadun[1], Brian Bush[1], Arthur Yip[1], Catherine Ledna[1], Laura Vimmerstedt[1], Kara Podkaminer[2], Ookie Ma[2]

[1] National Renewable Energy Laboratory, Golden, CO, USA.
[2] U.S. Department of Energy, Washington, D.C., USA
[*] Corresponding author: Matteo.Muratori@NREL.gov

Supplementary Information

S1. Input variables

We define an *input variable* as a single exogenous input that can vary over time in TEMPO (e.g., gasoline price), and *input variable levels* represent different assumptions of trajectories for each input variable (e.g., a static gasoline price of $\$3\ \text{gal}^{-1}$). These variables represent changes from the baseline across categories of technology, behavior, and policy.

Table S1. Summary of input variables. Inputs are derived in combination from targeted expert elicitation and various literature. For single-variable scenarios, all variables use the baseline level except for the isolated variable of interest. For multivariable scenarios, we use a Sobol sequence algorithm in R[1] to choose quasi-random, low-discrepancy sequences to sample a quasi-uniform distribution of variable combinations. "AEO" refers to inputs in the baseline that match EIA's 2019 Annual Energy Outlook[2]. "NHTS" refers to inputs derived from the 2017 National Household Travel Survey[3]. "FAF" refers to the Freight Analysis Framework[4]. "ATB" refers to inputs derived from the Annual Technology Baseline study[5] with ATB "Con," "Mid," and "Adv" referring to the constant, mid, and advanced scenarios, respectively. BEV = battery electric vehicle; DCFC = direct current fast charge; FCEV = fuel cell electric vehicle; GGE = gasoline gallon equivalent; LDV = light duty vehicle; MaaS = mobility as a service; MHD = medium-heavy duty; PHEV = plug-in electric vehicle; WTW = well-to-wheel; ZEV = zero-emissions vehicle.

**Input Variable**	**Input Description**	**Input Variable Levels**	**Input Category**
WTW biogas emissions	Well-to-wheel biofuel emissions reductions	Base: Current (−50% from gas)<br>L2: −70% from gas<br>L3: −75% from gas	Technology
WTW biodiesel emissions		Base: Current (−50% from diesel)<br>L2: −70% from diesel<br>L3: −140% from diesel	Technology
WTW biojet emissions		Base: Current (−50% from jet)<br>L2: −75% from jet<br>L3: −140% from jet	Technology
Light-duty BEV costs	Cost trajectories of light-duty battery-electric vehicles including batteries	Base: AEO<br>L2: ATB Mid<br>L3: Constant<br>L4: ATB Adv ($80/kWh)<br>L5: $40/kWh<br>L6: $60/kWh	Technology
MHD BEV costs	Cost trajectories of medium-/heavy-duty battery-electric vehicles including batteries	Base: AEO<br>L2: ATB Mid<br>L3: Constant<br>L4: ATB Adv ($80/kWh)<br>L5: $40/kWh<br>L6: $60/kWh	Technology

Light-duty FCEV costs	Cost trajectories of light-duty fuel cell electric vehicles	Base: AEO<br>L2: ATB Mid<br>L3: Constant<br>L4: ATB Adv	Technology
MHD FCEV costs	Cost trajectories of medium-/heavy-duty fuel cell electric vehicles	Base: AEO<br>L2: ATB Mid<br>L3: Constant<br>L4: ATB Adv	Technology
DCFC charging power	Power of public fast charging (higher = faster recharge)	Base: 125 kW<br>L2: 150 kW<br>L3: 350 kW	Technology
Light-duty PHEV costs	Cost trajectories of light-duty plug-in hybrid electric vehicles	Base: AEO<br>L2: ATB Mid<br>L3: Constant<br>L4: ATB Adv ($80/kWh)<br>L5: $40/kWh<br>L6: $60/kWh	Technology
Light-duty combustion-only powertrain costs	Cost trajectories of light-duty combustion-only vehicles (gasoline, natural gas)	Base: AEO<br>L2: ATB Mid	Technology
MHD vehicle combustion-only powertrain costs	Cost trajectories of medium-/heavy-duty combustion-only vehicles (gasoline, diesel, natural gas)	Base: AEO<br>L2: ATB Mid	Technology
Light-duty BEV fuel economies	Fuel economy trajectories of light-duty battery-electric vehicles	Base: AEO<br>L2: ATB Mid<br>L3: ATB Con<br>L4: ATB Adv	Technology
Light-duty PHEV fuel economies	Fuel economy trajectories of light-duty plug-in hybrid electric vehicles	Base: AEO<br>L2: ATB Mid<br>L3: ATB Con<br>L4: ATB Adv	Technology
Light-duty FCEV fuel economies	Fuel economy trajectories of light-duty fuel cell electric vehicles	Base: AEO<br>L2: ATB Mid<br>L3: ATB Con<br>L4: ATB Adv	Technology
LDV combustion-only fuel economies	Fuel economy trajectories of light-duty combustion-only vehicles (gasoline, natural gas)	Base: AEO<br>L2: ATB Mid<br>L3: ATB Con<br>L4: ATB Adv	Technology
MHD BEV fuel economies	Fuel economy trajectories of medium-/heavy-duty battery-electric vehicles	Base: AEO<br>L2: ATB Mid<br>L3: ATB Con<br>L4: ATB Adv	Technology
MHD FCEV fuel economies	Fuel economy trajectories of medium-/heavy-duty fuel cell electric vehicles	Base: AEO<br>L2: ATB Mid<br>L3: ATB Con<br>L4: ATB Adv	Technology
MHD vehicle combustion-only fuel economies	Fuel economy trajectories of medium-/heavy-duty combustion-only vehicles (gasoline, diesel, natural gas)	Base: AEO<br>L2: ATB Mid<br>L3: ATB Con<br>L4: ATB Adv	Technology
Change in MaaS cost	Change in cost per mile to user for Mobility-as-a-Service	Base: Current<br>L2: −50%<br>L3: +50%<br>L4: −25%<br>L5: +25%	Technology

Change in MaaS travel time	Change in travel time to user for Mobility-as-a-Service	Base: Current<br>L2: −50%<br>L3: +50%<br>L4: −25%<br>L5: +25%	Technology
Carbon price by 2030	Carbon price in gradually phased (s-shape) reaching targeted cost per metric ton by 2030	Base: none<br>L2: $500/ton<br>L3: $100/ton<br>L4: $200/ton<br>L5: $300/ton<br>L6: $400/ton	Policy
Biogas fuel blend	Fuel blend by volume for biogasoline (ethanol)	Base: Current<br>L2: 15%	Policy
Biodiesel fuel blend	Fuel blend by volume for biodiesel	Base: Current<br>L2: 25%<br>L3: 5%<br>L4: 10%<br>L5: 15%<br>L6: 20%	Policy
Biojet fuel blend	Fuel blend by volume for biojet fuel	Base: Current<br>L2: 100%<br>L3: 20%<br>L4: 60%<br>L5: 80%	Policy
100% light-duty ZEV sales mandate	Year of mandate (immediate) requiring 100% light-duty ZEV sales (restricts sale of gasoline, natural gas, and non-plug-in hybrid vehicles, but allows plug-in hybrids vehicles)	Base: Never<br>L2: 2030<br>L3: 2035<br>L4: 2040<br>L5: 2045	Policy
100% MHD ZEV sales mandate	Year of mandate (with 5-year linear phase-in) requiring 100% medium-/heavy-duty ZEV sales (restricts sale of gasoline, diesel, natural gas vehicles)	Base: Never<br>L2: 2035<br>L3: 2040<br>L4: 2045	Policy
Mean household vehicle ownership	Mean number of personal vehicles owned by a household	Base: NHTS<br>L2: −50%<br>L3: −40%<br>L4: −30%	Behavior
LDV occupancy	Mean occupancy of light-duty vehicles	Base: NHTS<br>L2: −25%<br>L3: −10%<br>L4: +10%<br>L5: +25%<br>L6: +50%	Behavior
LDV non-cost preferences	Year and type of convergence for calibrated light-duty vehicle logit parameters to account for non-cost attributes not explicitly modeled (parity means technologies compete only on cost)	Base: AEO<br>L2: 2030 ZEV parity<br>L3: 2040 ZEV parity<br>L4: 2030 ZEV parity, 2025 hybrid parity<br>L5: 2040 ZEV parity, 2025 hybrid parity	Behavior

Value of time charging	Travelers' value of time while charging a battery. It is assumed that other refueling (gasoline, hydrogen) are quick enough to be negligible.	Base: Current<br>L2: +100%<br>L3: 0<br>L4: −50%	Behavior
Mean household trip frequency	Mean number of trips taken by household type (see Section S3.4 for more details)	Base: NHTS<br>L2: +10%<br>L3: −15%<br>L4: +5%<br>L5: −7.5%	Behavior
Mean household trip length	Mean length of trips taken by household type (see Section S3.4 for more details)	Base: NHTS<br>L2: +10%<br>L3: −10%<br>L4: −5%<br>L5: +5%	Behavior
Freight demand growth	Trajectory of growth for freight demand (in tonne-miles; see Section S3.4 for more details)	Base: AEO<br>L2: +20%<br>L3: −20%<br>L4: +10%<br>L5: −10%	Behavior
Mean freight trip length	Mean length of trips taken by freight segment with total freight demand held constant (this effectively increases demand for shorter trips while decreasing demand for longer trips, or vice versa; see Section S3.4 for more details)	Base: FAF<br>L2: +50%<br>L3: −50%<br>L4: +25%<br>L5: −25%	Behavior
Freight cost payback	Maximum number of years for freight operators to payback costs for medium-/heavy-duty freight vehicles	Base: 2 years<br>L2: 4 years<br>L3: 8 years<br>L4: 15 years	Behavior
Non-LDV occupancy (10% MaaS deadhead)	Non-light-duty passenger mode occupancy (e.g., transit, air)	Base: Current<br>L2: −50%<br>L3: +100%<br>L4: −25%<br>L5: +25%<br>L6: +50%	Behavior
Fossil fuel prices	Change in fossil fuel prices phased in (s-shape) from 2019 baseline	Base: AEO<br>L2: −50%<br>L3: +50%<br>L4: −25%<br>L5: +25%	Technology & Policy
Electricity prices	Change in electricity prices phased in (s-shape) from 2019 baseline	Base: AEO<br>L2: \$0.27/kWh residential & DCFC, +100% commercial<br>L3: \$0.06/kWh residential, −80% commercial	Technology & Policy
Hydrogen prices	Change in hydrogen prices to consumers phased in (s-shape) from 2019 baseline	Base: Current (\$16.24/GGE)<br>L2: \$7/GGE by 2030, \$3.40/GGE by 2040<br>L3: \$7/GGE by 2025, \$4/GGE by 2030<br>L4: \$10/GGE by 2040<br>L5: \$8/GGE by 2040<br>L6: \$6/GGE by 2040	Technology & Policy
Households with home charging	Percent of households with Level 2 charging capability (perceived)[6]	Base: 11%<br>L2: 31%<br>L3: 47%<br>L4: 60%<br>L5: 75%	Technology & Policy

Household hydrogen refueling access	Percent of households with access to hydrogen refueling stations for light-duty vehicles	Base: 0%<br>L2: 100%<br>L3: 50%	Technology & Policy
Freight hydrogen refueling access	Percent of freight fleets with access to hydrogen refueling stations for medium-/heavy-duty vehicles	Base: 5%<br>L2: 100%<br>L3: 50%	Technology & Policy
Household bus access	Households with access to bus transit (public and commuter)	Base: Current<br>L2: −50%<br>L3: +50%	Technology & Policy
Household metro rail access	Households with access to metro rail transit (light rail, heavy rail)	Base: Current<br>L2: −50%<br>L3: +50%	Technology & Policy
Household commuter rail access	Households with access to commuter rail transit	base: Current<br>L2: −50%<br>L3: +50%	Technology & Policy
Household MaaS access	Households with access to Mobility-as-a-Service (e.g., on-demand ride-share)	Base: Current<br>L2: −50%<br>L3: +50%	Technology & Policy
Change in on-road fuel economies	Change in all vehicles on-road fuel economies (e.g., due to congestion; both passenger and freight sector on-road vehicles)	Base: Baseline<br>L2: −25%<br>L3: +25%<br>L4: −15%<br>L5: +15%	Technology & Policy
Change in on-road travel time	Change in all vehicles' on-road travel times (e.g., due to congestion; both passenger and freight sector on-road vehicles)	Base: Baseline<br>L2: +25%<br>L3: −25%<br>L4: +15%<br>L5: −15%	Technology & Policy
Household public charging access	Household access to Level 2 public charging for electric vehicles	Base: 50%<br>L2: 0%<br>L3: 100%	Technology & Policy
LDV retirement rates	Rate of light-duty vehicle retirement (i.e., removed from fleet entirely)	Base: Current<br>L2: −50%<br>L3: +50%<br>L4: −25%<br>L5: +25%	Technology & Behavior

S2. TEMPO baseline scenario

The baseline TEMPO scenario is aligned with EIA's Annual Energy Outlook (AEO) 2019 Reference Scenario. We match to the 2019 AEO Reference for several reasons: (1) to have a consistent initial year with TEMPO sourced demand data from the 2017 National Household Travel Survey[3] and Freight Analysis Framework[4]; (2) to match a scenario that assumes no significant progress in technology, behavior, or policy; (3) to avoid a scenario that is impacted by significant short-term changes from COVID (long-term changes that results from COIVD are still considered). This TEMPO scenario is used in this study as the "baseline" to serve as a reference point of comparison for sensitivity and uncertainty analyses, as it ensures our reference point for comparisons assumes no significant progress in technology, behavior, or policy (e.g., 13% vehicle electrification in 2050 and continued petroleum dominance). AEO provides comprehensive and widely accepted projections of energy supply and demand including fuel prices, travel demand, vehicle stock, and energy use in the United States through 2050 based on EIA's National Energy Modeling System. By supplementing some exogenous TEMPO inputs with consistent AEO-based

assumptions including fuel costs, vehicle cost, and fuel economy projections, and by calibrating the TEMPO technology adoption logit formulation, TEMPO is calibrated to match 2017 domestic transportation energy use estimates by mode, technology, and fuels reported by EIA. Comparing energy use in future years shows consistent alignment over time, with TEMPO energy use by mode and fuel closely matching AEO projections (Figure S1).

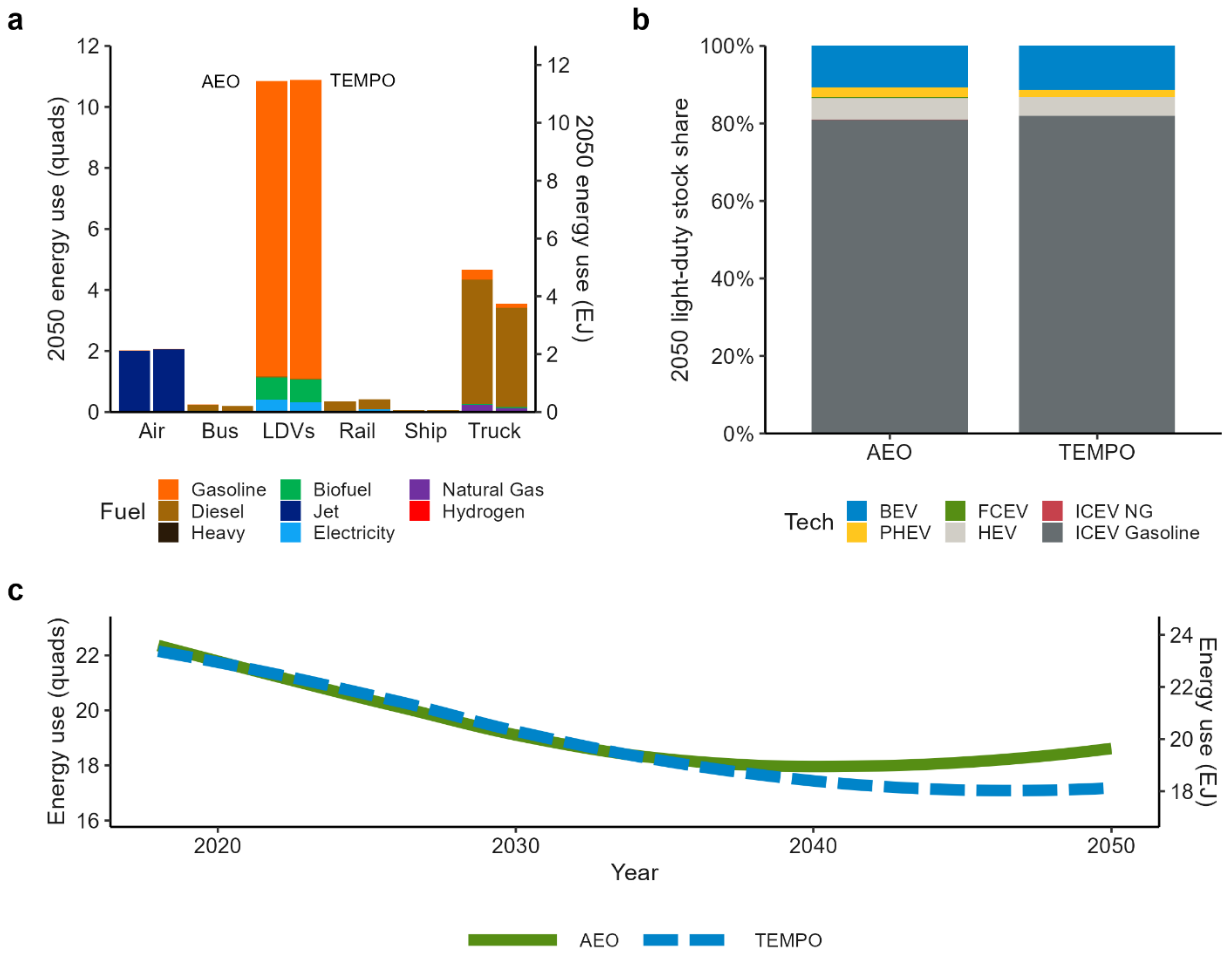

Figure S1. Overview of the TEMPO baseline compared to the 2019 Annual Energy Outlook (AEO) Reference Case[2]. Panel (a) shows the 2050 energy use comparison, panel (b) shows the 2050 light-duty stock share comparison, and panel (c) compares transport energy use of both models between 2018 and 2050. Note in (c) that the y-axis is truncated to start at 16 quads to magnify the closely matching total sector energy use of the models.

S3. Travel demand changes

There is great uncertainty on possible travel demand changes driven by land use decisions and urban design (e.g., more compact polycentric cities with mixed land use patterns can reduce the distance between where people live, work, and pursue leisure activities) or transportation demand management (TDM) solutions, such as congestion pricing, gas or carbon taxes, higher parking fees, incentivized carpooling, or increased access to public and/or active transit. Empirical evidence and modeled scenarios often point to a collection of strategies to achieve the most impact from TDM. Relatedly, one of the most consistent findings is that more sprawling development increases per-capita energy consumed for travel[7–10], indicating that changes to urban form could lead lower emissions footprints. However, recent research has noted that while a relationship between density and travel demand exists, it is complex and a multitude of other more direct factors are at play, including the distribution of housing within a region, accessibility to jobs and opportunities, and household income or travel budgets[11,12].

S3.1. Changes to passenger travel demand changes

Most studies that have investigated TDM focus on passenger travel in a single city or region and observe or simulate a selection of strategies that focus on reductions in private car use, mode shift, or emissions reductions. Rarely do studies attempt to quantify changes in overall travel demand at a regional or national level, but a few have quantified impacts from TDM and urban form. Brownstone and Golob (2009) found a 5% change in household vehicle miles traveled (VMT) attributed to density alone using travel data from California[8]. Rodier and Johnston (1997) found that pricing car travel (increased costs for parking, gas, and congestion pricing) combined with a light rail transit expansion in Sacramento could reduce VMT in the region by 10%[13]. Bento et al. (2005) found that factors such as population centrality, the job–housing balance, urban form, and road density strongly impact household VMT and could achieve reductions of 25%[14]. A meta-review of U.S.-focused literature suggests doubling residential density in a region may lower household VMT by 5%–12%, and when coupled with other land use factors such as mixed use and increased accessibility, averages of 3%–20% reduction could be achieved and could reach as high as 25%[15]. Another meta-review of literature found that up to 25% reduction in land-based mobility emissions may be possible with benefits to well-being from avoiding excess travel (i.e., more telework, compact city design, and more active travel) and/or shifting travel (i.e., shared/pooled mobility, improved transit, and improved freight logistics); however it is unclear how avoiding and shifting would compound in reduction potential[16]. A large study in California shows that design solutions, better access to clean modes of travel, and behavioral changes supported by pricing and demand management policy implementation require multidecadal transitions, and by 2050 these strategies could approximately reduce miles traveled, and thus transportation emissions, up to 15%[17].

S3.2. Changes to freight travel demand

TDM for freight is less explored in literature. Freight trip generation models typically have weak exploratory power to accurately predict freight demand based on exogenous factors[18]. Like the passenger sector, one of the most consistent findings is that lower-density development may induce higher relative freight demand. Holguín-Veras et al. (2016) compared four metropolitan regions

from large to small and estimated that larger metros have fewer freight trips generated per capita and per establishment, but more trips per employed persons[19]. Retail was the most significant sector associated with freight trip demand (35%–44% of total share), and small businesses (nine employees or fewer) contributed 45%–60% of freight trip demand. Dablanc and Rakotonarivo (2010) discuss the spatial deconcentration of logistics terminals (logistics sprawl) in metropolitan Paris over four decades and find that it caused a mere 0.2% increase in freight emissions. Opportunities for freight TDM include off-hour delivery, last-mile consolidation of freight from multiple suppliers, delaying nonessential deliveries, and other logistic optimization solutions. Transport for London found success with management programs to impact surges in transport during the 2012 Olympics, especially to help manage freight planning and delivery[20]. Transport for London surveys found 58% of freight operators and 57% of businesses made some change to operations, and vehicle traffic from commercial vehicles reduced 10% during the Olympic periods relative to expected demand.

S3.3. COVID-19 and the future of travel demand

While it is not clear what feasible potential for travel demand reduction exists at a national level, the recent global COVID-19 pandemic has shed light on what is likely an upper limit under *extreme* circumstances. In the United States, Apple Mobility Trends (changes in routing requests) showed a drop of 50% for driving and 80% for transit during initial lockdowns in March and April 2020 relative to January 2020 trends[21]. After initial lockdowns were over (May 2020), routing requests for driving returned to normal, but transit requests did not return to normal until over a year later in winter 2021. However, many major transit systems across the United States are still lagging behind pre-pandemic ridership levels[22–24]. Data from the Bureau of Transportation Statistics show similar trends; around spring 2019, there were approximately 1.4 billion daily trips in the United States. During initial COVID-19 lockdowns, total trips dropped to a low of 0.84 billion daily trips and fluctuated around 0.90 billion daily trips until early spring 2021 (when widespread vaccine rollouts begun)[25]. Since spring 2021, daily trips have remained relatively stable at a potentially slightly lower "new normal" of 1.3 billion trips per day. While total numbers of trips taken almost realigned with pre-pandemic levels by the end of 2021, data show changes in travel mode choices and an increase in shorter trips[26]. MTA (New York City) and WMATA (Washington, D.C.) bus and rail ridership in March 2022 were still approximately 50% lower compared to 2019, and the San Francisco BART was 70% lower. Domestic flight departures were 10% lower in 2022 compared to 2019 (30% for international flights). Research is nascent and ongoing, but these changes are speculated to be due to increases in virtual presence (especially telework)[27], but long-term impacts on transit remain uncertain. Other technology advancements are also impacting travel demand, further increasing uncertainty. Virtual presence has been shown to reduce travel demand even before COVID-19[28]. On the other hand, vehicle automation has been shown to possibly increase travel demand, especially trip length[29].

S3.4. Modeling travel demand changes

We evaluate changes to travel demand under two passenger and two freight scenarios with input variables that exogenously force more or less travel by shifting underlying distributions of travel demand. Given the complexity and high uncertainty for the evolution of travel demand, the scenarios are meant to capture potential aggregate changes to demand as a result of multiple factors

impacting urban design, consumer options and choices, and needs to travel, as well as elucidate potential for decarbonization due to shifts in travel behavior. We assess bounds of future changing travel demand in two forms to capture structural shifts in demand in response to technology, policy, or behavior not otherwise explicitly simulated in TEMPO from the various mechanisms outlined in this Supplementary Information: (1) changes in frequency of trips over time and (2) changes in length of trips over time. Given the extreme but unrealistic travel demand reductions during COVID-19 lockdowns and the more moderate evidence for TDM and shifts in behavior that impact travel demand, we evaluate uncertainty in the frequency of trips changing between −15% and +10% and the length of trips changing between −15% and +10% by 2050. The compounded effect is a change in travel demand ranging between −28% and +21% by 2050.

For passenger (household-based) travel, we fit against the National Household Travel Survey for 60 unique types of households across three statistically significant dimensions: urbanity (more rural regions have more long-distance trips and longer trip lengths on average), income (travel budget), and number of drivers in a household. There are two types of passenger demand changes we consider: *mean household trip frequency* and *mean household trip length*, which change the mean frequency and mean length of trips demanded by household type. We shift the mean of fitted travel demand distributions by a factor (levels listed in Table S1) across all household types. An example of one distribution for a household type with different input data assumptions for *mean household trip length* is shown in Figure S2(a).

There are two types of freight demand changes we consider: *freight demand growth* (changes to the future growth trajectories of total tonne-miles) and *mean freight trip length* (changes to the distances of shipments for tonne-miles). Changes to growth of demanded freight assume no change in the distribution of tonne-miles by distance bin (unless combined with the freight demand scenario in uncertainty simulations); thus, in isolation, total demand increases or decreases directly due to more overall demand at the same distributions of shipment distances. On the other hand, changes to the distance of shipments assume that the tonnes of demand is unchanged in aggregate, but is shipped longer or shorter distances on average. As a result, this changes total tonne-miles demanded because distances become shorter or longer while tonnage stays constant (tonne-miles = tonnes × miles). The input variable *mean freight trip length* and scenarios for changing freight trip lengths are shown in Figure S2(b).

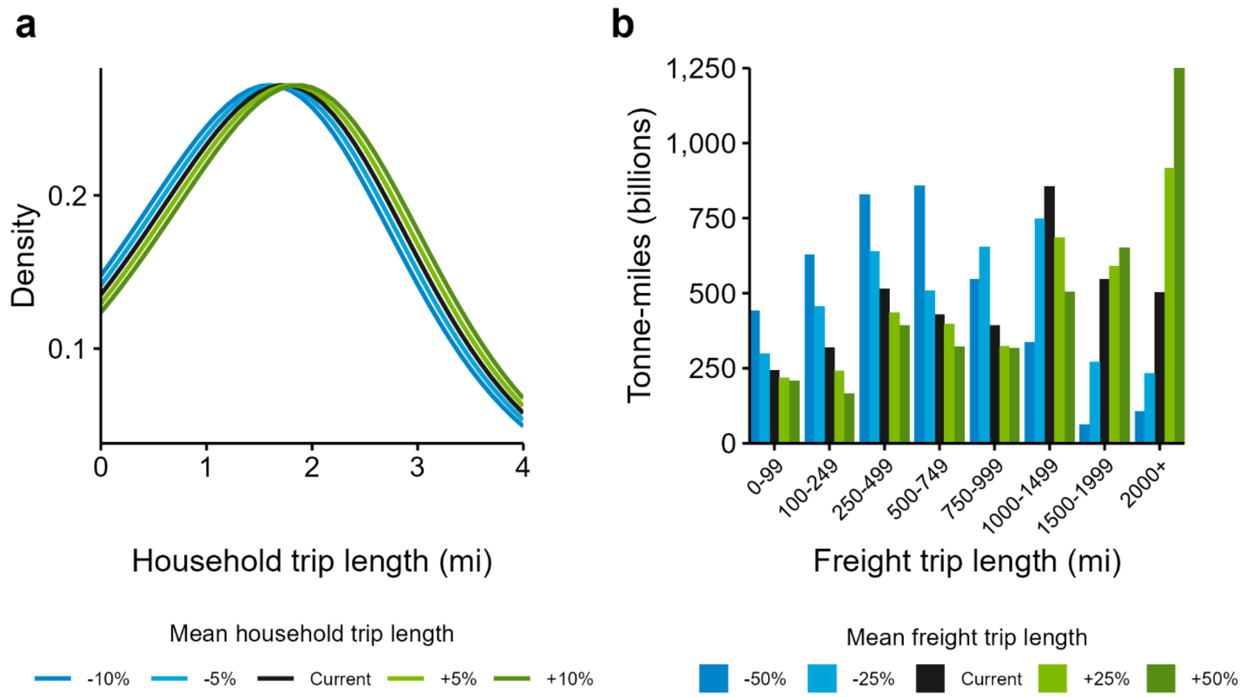

Figure S2. (a) Passenger and (b) freight inputs for changes to trip length distributions. Panel (a) shows an example change to the trip length distribution for one household type (low-income, rural household with no drivers, one of 60 types of households each with uniquely fitted trip length distributions from 2017 National Household Travel Survey data). Note that household trip lengths extend past 4 miles, but the axis is truncated for visibility. Panel (b) shows freight demand for the input year of 2017. These distributions are held constant through 2050 in the baseline, but changes to these inputs are phased in from 2020 to 2050.

S4. Supplementary figures

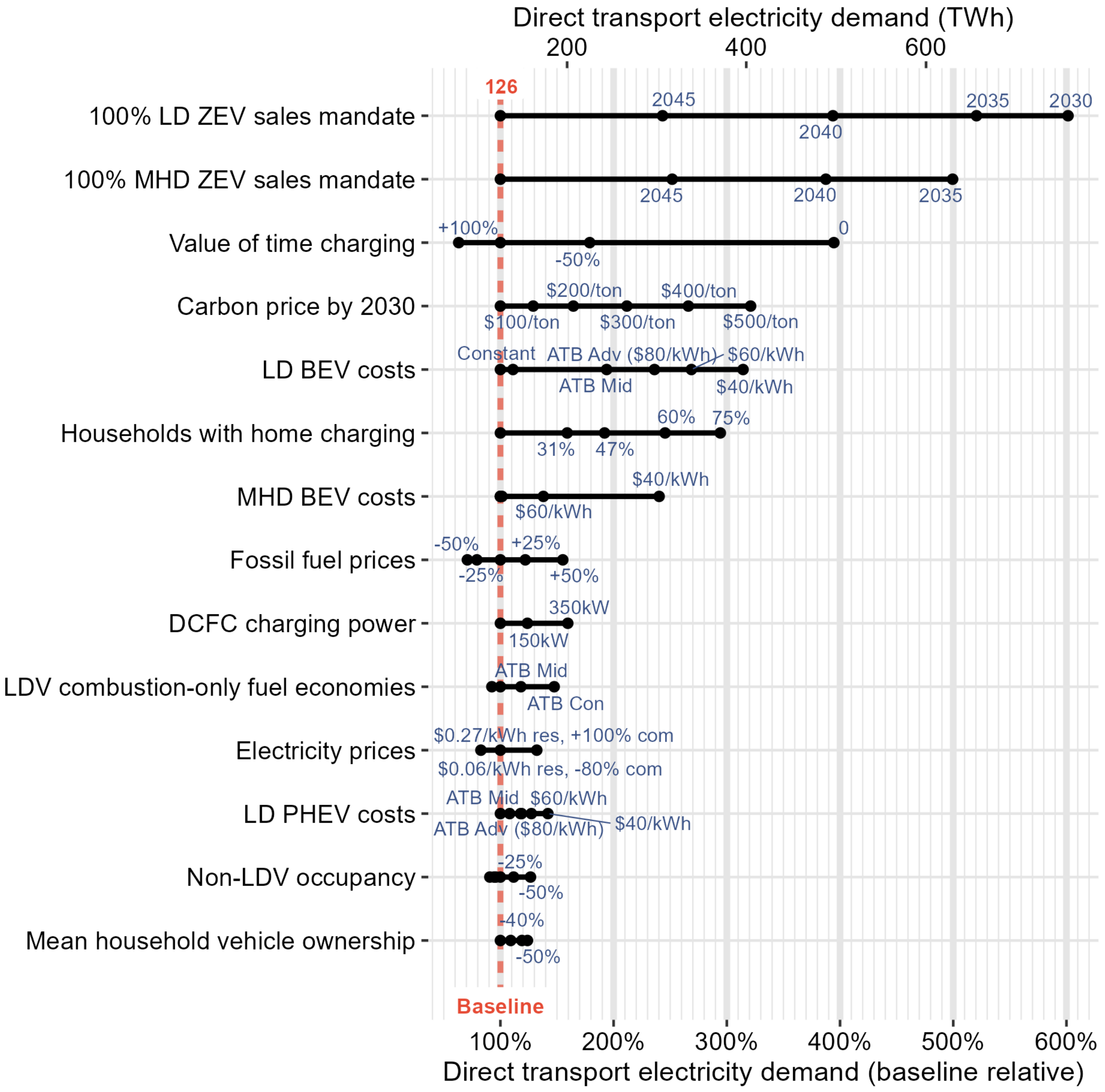

Figure S3. Isolated variable impacts on direct transport electricity demand. Direct electricity demand includes use from electric vehicles including passenger rail but excludes any electricity consumed in producing hydrogen or biofuels. Input categories with maximum electricity impacts less than 20 TWh in 2050 are excluded. The base case of transportation electricity demand in 2050 is 126 TWh. Battery electric vehicle (BEV) costs and fuel economies are based on the 2020 Annual Technology Baseline (ATB) study[5], with ATB "Con," "Mid," and "Adv" referring to the constant, mid, and advanced scenarios, respectively, with two additional scenarios of battery cost reduction assumptions ($60 kWh^{-1} and $40 kWh^{-1} by 2050). LD = light duty; MHD = medium-heavy duty; ZEV = zero-emissions vehicle; PHEV = plug-in electric vehicle; DCFC = direct current fast charge.

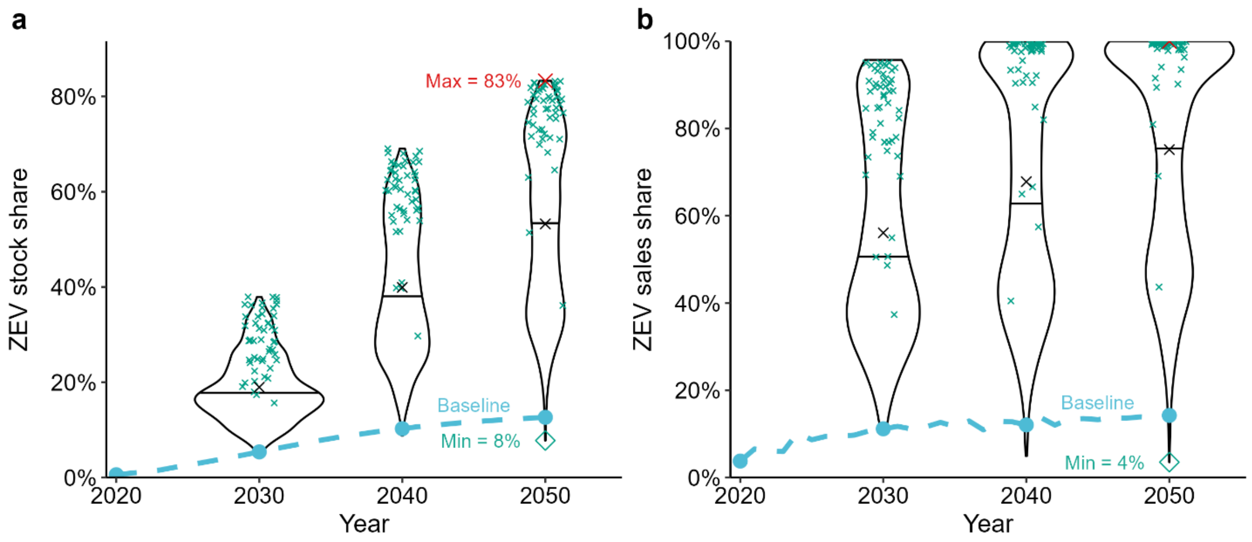

Figure S4. (a) Decadal U.S. zero emission vehicle (ZEV) stock and (b) sales shares across 2,000 multivariable simulations of TEMPO. Deep decarbonization scenarios (N=50 in each year) are denoted with green "x" marks, and the mean share is a larger black "x." These results do not indicate the likelihood of outcomes.

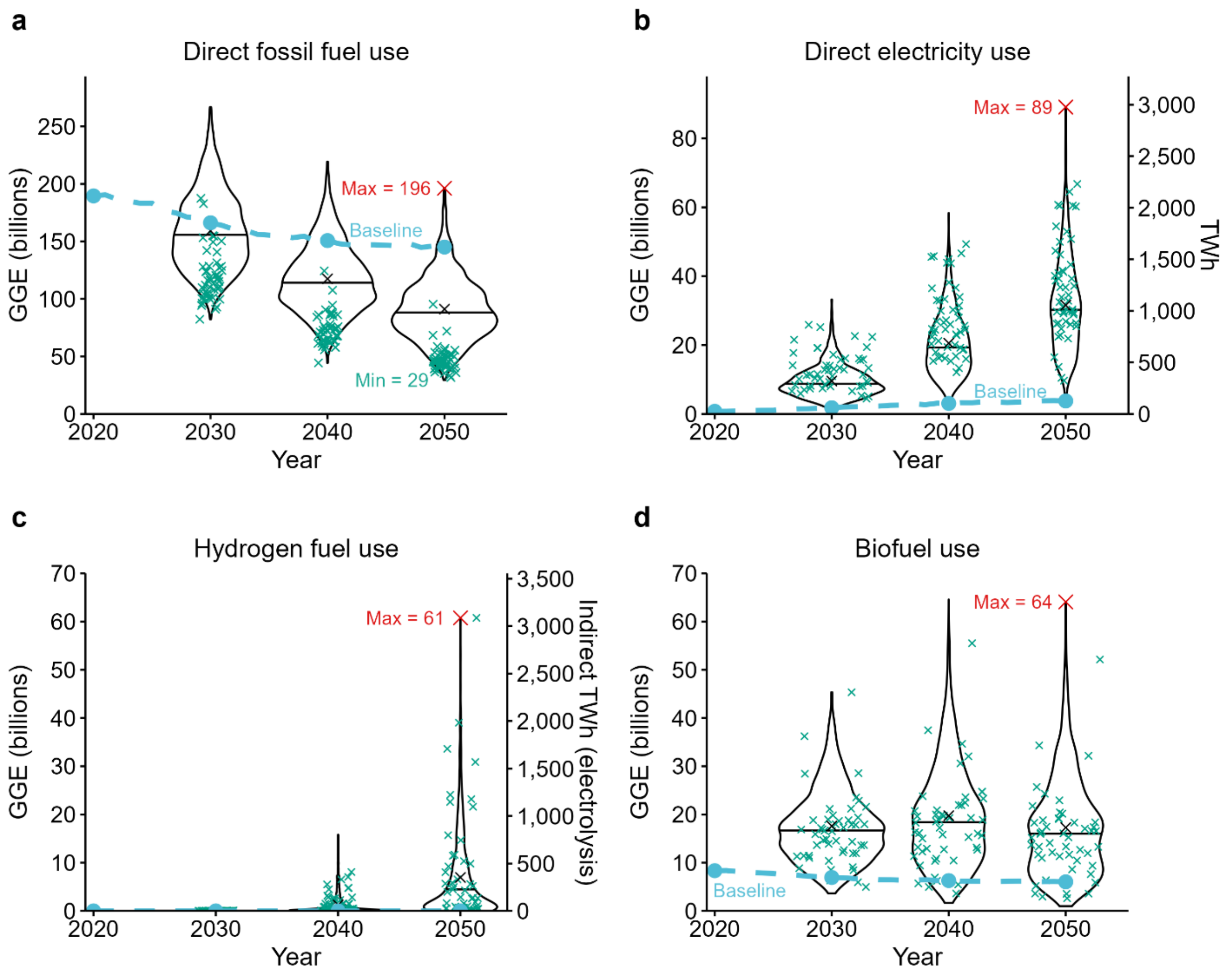

Figure S5. Decadal U.S. domestic mobility energy use by type across 2,000 multivariable simulations of TEMPO. Panel (a) shows direct fossil fuel consumption (use phase), panel (b) shows direct electricity consumption (electricity used from batteries and electrified rail but excludes electrolysis-made hydrogen in fuel cells), panel (c) shows hydrogen fuel consumption, and panel (d) shows biofuel consumption. Deep decarbonization scenarios (N=50 in each year) are denoted with green "x" marks, and the mean share is a larger black "x." These results do not indicate the likelihood of outcomes. GGE = gasoline gallon equivalent.

Supplementary References

1. Kuo, F. & Joe, S. SobolSequence: Sobol Sequences with Better Two-Dimensional Projections. Preprint at (2017).
2. EIA, (Energy Information Administration). *Annual Energy Outlook 2019 with projections to 2050*. https://www.eia.gov/outlooks/aeo/pdf/aeo2019.pdf (2019).
3. FHWA, (Federal Highway Administration). 2017 National Household Travel Survey. http://nhts.ornl.gov (2017).
4. FHWA, (Federal Highway Administration). Freight Analysis Framework - 2017. (2019).
5. NREL, (National Renewable Energy Laboratory). *2020 Transportation Annual Technology Baseline*. https://atb.nrel.gov/ (2020).
6. Ge, Y., Simeone, C., Duvall, A. & Wood, E. *There's No Place Like Home: Residential Parking, Electrical Access, and Implications for the Future of Electric Vehicle Charging Infrastructure*. (2021) doi:10.2172/1825510.
7. Nichols, B. G. & Kockelman, K. M. Life-cycle energy implications of different residential settings: Recognizing buildings, travel, and public infrastructure. *Energy Policy* **68**, 232–242 (2014).
8. Brownstone, D. & Golob, T. F. The impact of residential density on vehicle usage and energy consumption. *J Urban Econ* **65**, 91–98 (2009).
9. Norman, J., MacLean, H. L. & Kennedy, C. A. Comparing High and Low Residential Density: Life-Cycle Analysis of Energy Use and Greenhouse Gas Emissions. *J Urban Plan Dev* **132**, 10–21 (2006).
10. Kenworthy, J. R. & Laube, F. B. Patterns of automobile dependence in cities: an international overview of key physical and economic dimensions with some implications for urban policy. *Transp Res Part A Policy Pract* **33**, 691–723 (1999).
11. Ewing, R. *et al.* Testing Newman and Kenworthy's Theory of Density and Automobile Dependence. *J Plan Educ Res* **38**, 167–182 (2018).
12. Chao, L. & Qing, S. An empirical analysis of the influence of urban form on household travel and energy consumption. *Comput Environ Urban Syst* **35**, 347–357 (2011).
13. Rodier, C. J. & Johnston, R. A. Travel, Emissions, and Welfare Effects of Travel Demand Management Measures. *Transportation Research Record: Journal of the Transportation Research Board* **1598**, 18–24 (1997).
14. Bento, A. M., Cropper, M. L., Mobarak, A. M. & Vinha, K. The Effects of Urban Spatial Structure on Travel Demand in the United States. *Review of Economics and Statistics* **87**, 466–478 (2005).

15. National Research Council. *Driving and the Built Environment: The Effects of Compact Development on Motorized Travel, Energy Use, and CO2 Emissions -- Special Report 298*. (The National Academies Press, 2009). doi:10.17226/12747.

16. Creutzig, F. *et al.* Demand-side solutions to climate change mitigation consistent with high levels of well-being. *Nat Clim Chang* **12**, 36–46 (2022).

17. Brown, A. L. *et al. Driving California' s Transportation Emissions to Zero*. (2021). doi:10.7922/G2MC8X9X.

18. Holguín-Veras, J. *et al.* Transferability of Freight Trip Generation Models. *Transportation Research Record: Journal of the Transportation Research Board* **2379**, 1–8 (2013).

19. Holguín-Veras, J., Sánchez-Díaz, I. & Browne, M. Sustainable Urban Freight Systems and Freight Demand Management. *Transportation Research Procedia* **12**, 40–52 (2016).

20. Allen, J., Browne, M. & Woodburn, A. London Freight Data Report: 2013 Update. *Transport for London* 1–27 (2014).

21. Apple. Mobility Trends Reports. https://covid19.apple.com/mobility (2022).

22. MTA, (Metropolitan Transportation Authority). Day-by-day ridership numbers. https://new.mta.info/coronavirus/ridership (2022).

23. BART, (Bay Area Rapid Transit). Ridership Reports. https://www.bart.gov/about/reports/ridership (2022).

24. BTS, (Bureau of Transportation Statistics). Latest Weekly COVID-19 Transportation Statistics. https://www.bts.gov/covid-19/week-in-transportation#transit (2022).

25. BTS, (Bureau of Transportation Statistics). Daily Travel during the COVID-19 Public Health Emergency. https://www.bts.gov/daily-travel (2022).

26. EPA, (Environmental Protection Agency). *Inventory of U.S. Greenhouse Gas Emissions and Sinks: 1990 – 2014*. (2016).

27. Mouratidis, K. & Papagiannakis, A. COVID-19, internet, and mobility: The rise of telework, telehealth, e-learning, and e-shopping. *Sustain Cities Soc* **74**, 103182 (2021).

28. Shabanpour, R., Golshani, N., Tayarani, M., Auld, J. & Mohammadian, A. (Kouros). Analysis of telecommuting behavior and impacts on travel demand and the environment. *Transp Res D Transp Environ* **62**, 563–576 (2018).

29. Harb, M., Xiao, Y., Circella, G., Mokhtarian, P. L. & Walker, J. L. Projecting travelers into a world of self-driving vehicles: estimating travel behavior implications via a naturalistic experiment. *Transportation (Amst)* **45**, 1671–1685 (2018).
